# Can ship travel contain COVID-19 outbreak after re-opening: a Bayesian meta-analysis

**DOI:** 10.1017/S0950268823000821

**Published:** 2023-05-25

**Authors:** Chen-Yang Hsu, Jia-Kun Chen, Paul S. Wikramaratna, Amy Ming-Fang Yen, Sam Li-Sheng Chen, Hsiu-Hsi Chen, Chao-Chih Lai

**Affiliations:** 1 Institute of Epidemiology and Preventive Medicine, College of Public Health, National Taiwan University, Taipei, Taiwan; 2 Master of Public Health Program, National Taiwan University, Taipei, Taiwan; 3 Institute of Environmental and Occupational Health Sciences, College of Public Health, National Taiwan University, Taipei, Taiwan; 4 Independent Consultant, London, England; 5 School of Oral Hygiene, College of Oral Medicine, Taipei Medical University, Taipei, Taiwan; 6 Emergency Department of Taipei City Hospital, Ren-Ai Branch, Taipei, Taiwan

**Keywords:** Basic reproductive number, COVID-19, cruise ship, The Bayesian SEIR model, warship

## Abstract

Large gatherings of people on cruise ships and warships are often at high risk of COVID-19 infections. To assess the transmissibility of SARS-CoV-2 on warships and cruise ships and to quantify the effectiveness of the containment measures, the transmission coefficient (β), basic reproductive number (R_0_), and time to deploy containment measures were estimated by the Bayesian *Susceptible-Exposed-Infected-Recovered* model. A meta-analysis was conducted to predict vaccine protection with or without non-pharmaceutical interventions (NPIs). The analysis showed that implementing NPIs during voyages could reduce the transmission coefficients of SARS-CoV-2 by 50%. Two weeks into the voyage of a cruise that begins with 1 infected passenger out of a total of 3,711 passengers, we estimate there would be 45 (95% CI:25-71), 33 (95% CI:20-52), 18 (95% CI:11-26), 9 (95% CI:6-12), 4 (95% CI:3-5), and 2 (95% CI:2-2) final cases under 0%, 10%, 30%, 50%, 70%, and 90% vaccine protection, respectively, without NPIs. The timeliness of strict NPIs along with implementing strict quarantine and isolation measures is imperative to contain COVID-19 cases in cruise ships. The spread of COVID-19 on ships was predicted to be limited in scenarios corresponding to at least 70% protection from prior vaccination, across all passengers and crew.

## Introduction

Large gatherings of people in semiconfined settings, such as on cruise ships and warships, are often at high risk of infections [1]. Previous studies have already shown high attack rates of COVID-19 on cruise ships [2, 3], where the complex and frequent movements of passengers and high levels of direct and indirect personal contact facilitate the spread of the virus [4]. The higher population density in these settings renders them susceptible to the quick spread of pathogens harbored in the respiratory tract and digestive lumen [5–7].

The first cruise ship to have a major Severe Acute Respiratory Syndrome Coronavirus (SARS-CoV-2) outbreak on board was the *Diamond Princess* in February 2020 [8] in the wake of the original community-acquired outbreak identified in China in December 2019 [9, 10]. The first major SARS-CoV-2 outbreak on a naval ship was onboard the *Theodore Roosevelt* aircraft carrier in March 2020. After these, at least 50 outbreaks occurred during cruise ship voyages, yielding more than 1,100 confirmed cases, and over 20 naval ships, resulting in 2,500 confirmed cases as of March 2021 [11, 12]. Based on these reports, we decided to conduct a systematic review and meta-analysis to elucidate whether the transmissibility of SARS-CoV-2 is heterogeneous across these outbreaks.

Around 30 million people were transported worldwide on cruise ships in 2019, up 6% from 28.2 million in 2018 [13]. Unfortunately, many cruise lines around the world have been suspended, with cruise ships unable to operate during the pandemic for fear of fostering large outbreaks of disease. It is therefore imperative to provide guidance for forestalling COVID-19 outbreaks on cruise ships, and indeed warships, once suspected cases are identified [14]. It is worthy of investigating whether the isolation of suspected cases on board is sufficient to control an outbreak, or if a return to port followed by quarantine on land is necessary, and how these containment measures are potentially affected by vaccination.

Hence, the aims of our study are two-fold. One is to estimate relevant parameters related to the transmissibility of SARS-CoV-2 and examine whether the transmissibility of SARS-CoV-2 on cruise ships and warships was consistent across ship-based outbreaks by pooling multiple data from all outbreaks occurring on board on the basis of meta-analysis. The other is to assess the effectiveness of NPIs (non-pharmaceutical interventions) given different coverage rates of vaccination.

## Materials and methods

The data on COVID-19 outbreaks on cruise ships and warships obtained from public messages [15–18] and the literature [19–25] was used to estimate the parameters of a compartmental model. The characteristics of COVID-19 outbreaks on cruise ships and warships are listed in Table 1. Detailed information about these outbreaks in the six ships enrolled in this study is provided in the Supplementary Table A1.

## The Bayesian DAG of meta-analysis with an SEIR model underpinning

To model the dynamics of COVID-19 evolution on each ship, a four-compartment model of susceptible-exposed-infectious-removed (SEIR) was applied. The compartmental model based on the characteristics of COVID-19 is shown in Supplementary Figure A1 with detailed relationships and notations. In brief, the subjects on board were divided into four compartments, namely individuals susceptible to SARS-CoV-2 (S(t)), those exposed and infected but not yet infectious (E(t)), those infectious (I(t)), and those removed from the infectious compartment (R(t)). The S(t), E(t), I(t), and R(t) denote the number of cases at time t. Importantly, we assume that infectious individuals can enter the removal (R) state either because they recover from infection in the traditional sense and become immune or because they are fully and perfectly isolated from the rest of the onboard subjects. Given an effective exposure, the SEIR model assumes that all subjects of the exposed state (E) progress to the infectious state (I). This model was used to evaluate the propagation of SARS-CoV-2 on warships and cruise ships. Based on a previous application of an SEIR model for COVID-19 clustered events on board [24], the propagation of COVID-19 outbreaks on each ship was found to be driven by the transmission coefficient (β), the reciprocal of the infectious period (α), and the recovery rate (σ). Important assumptions in our SEIR model are as follows. (1) All cases in the E state will move into the infectious state by definition because we want to model the undetectable cases for evaluating the effect of NPIs; therefore, all cases of the E state became detectable cases (I+R compartment) later. (2) The E state referred to cases that were initially infected but undetectable (unobserved). If these cases are identified by test or the presence of symptoms, they are then considered to be in an infectious state. A detectable case is defined as an individual who can be identified by a test or by the presence of symptoms during an outbreak. The ratio of symptomatic and pre-symptomatic cases is dependent on the testing strategy used. For example, both were included in the outbreak on *Greg Mortimer*, whereas only the symptomatic case was included in the outbreaks on the *Charles de Gaulle* and *Theodore Roosevelt* aircraft carriers. Therefore, the number of predicted observed cases (detectable cases) was the total number of persons in the I and R compartment. The number of total predicted cases, including undetectable and detectable cases, was the total number of persons in the E, I, and R compartment in this model. (3) We assumed similar transmission probability between symptomatic and asymptomatic cases. (4) Although some crews and passengers left the board in batches without contacting other people after landing, a close population was still considered during the period to stay on board. Therefore, their status still needed to be followed by their testing results. By solving the nonlinear ordinary differential equations of the SEIR model, the expected number of subjects at each compartment can be derived as a function of the three parameters. In addition, we define the time at which containment measures are successful as the time beyond which no member of the S class progresses to the E class. Therefore, the estimated shortest time of successful containment (no member of the S class progresses to the E class) was at the moment the number of final observed cases was within the credible interval (CI) of predicted total cases (E+I+R) in our model.

**Figure 1.**
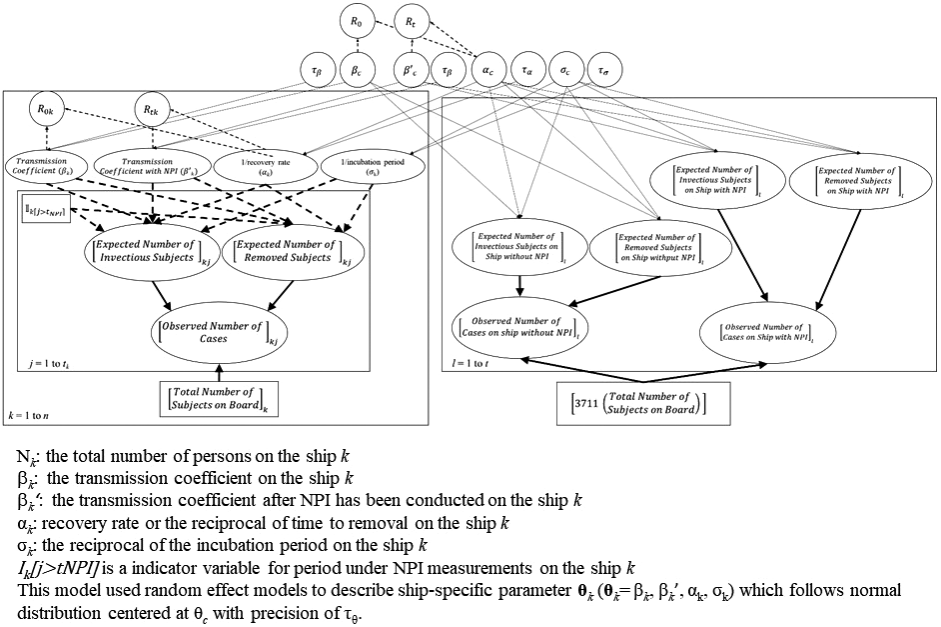
Bayesian DAG of the meta-analysis model for COVID-19 propagation on ships.

Stemming from the SEIR model mentioned above, we developed a Bayesian meta-analysis model depicting the COVID-19 outbreak for the six ships enrolled in this study. Figure 1 shows the Bayesian-directed acyclic graphic (DAG) of our meta-analysis model. The observed number of COVID-19 cases on ship *k* on Day *j* provides information on the expected numbers of infectious and removed subjects. This information, together with the fixed number of total onboard subjects for ship *k*, was used for the estimation of the three parameters, β*_k_*, α*_k_*, and σ*_k_*, embedded in the SEIR model. For ships that implemented NPIs, the impact on COVID-19 transmission was captured by the transmission coefficient β’*_k_.* The daily number of COVID-19 cases provides information on the evolution of the four compartments. A normal distribution was applied to capture the expected count of compartments S and E. Regarding the daily count of I and R, one among the normal, binomial, or Poisson distributions was selected depending on the convergence status, by using the trace plot of sampling history [24].

Using the Bayesian meta-analysis model, the information on COVID-19 propagation on each type of ship was integrated to derive the posterior information on the distribution of the three main parameters and the effectiveness of NPIs in containing COVID-19 outbreaks on ships. In addition, meta-analyses were also conducted on these outbreaks on cruise ships and warships. We used random effects on the log scale to capture the heterogeneity across ships for each parameter.

While informative priors for the two disease progression parameters β_k_ and *α_k_* were used to fit the mean duration from exposure to infectious and from infectious to recovered for 5.25 (95% CI: 4-7) and 7 (95% CI: 5-12) days, respectively, in the COVID-19 outbreak of the *Diamond Princess* cruise ship [10, 24, 26], non-informative priors were used for the transmission coefficient with (β_k_^’^) and without (β_k_) NPIs.

To cope with the uncertainty of full joint parameters related to these outbreaks, the Bayesian Markov Chain Monte Carlo (MCMC) was applied to estimate the transmission coefficients, recovery, and incubation rates, and their 95% CI of COVID-19 outbreaks on each ship. The posterior distributions of the common transmission coefficient with (β_c_^’^) and without (β_c_) NPIs, and the two disease progression parameters (α*_c_*, and σ*_c_*) were also derived from the Bayesian meta-analysis model.

With this integrated information on COVID-19 propagation, scenarios of future outbreaks on cruise ships under different conditions, such as with or without the implementation of NPIs or with varying levels of vaccine protection for onboard subjects, can be predicted (right plate, Figure 1). Specifically, we envisaged a cruise ship of the size of the *Diamond Princess* with a total of 3,711 crews and passengers on board heading for a 14-day voyage, with a single person in the infectious state (I) on initiation of the voyage (Day 0), who was then discovered on Day 7 of the voyage. We then considered how the imposition of NPIs at that point would affect the subsequent size of the outbreak, and additionally assessed the impact of 0%, 10%, 30%, 50%, 70%, and 90% of the ship’s population having been previously successfully vaccinated against SARS-CoV-2, where the influence of vaccine protection was captured by a reduction in transmission coefficient as β_c_*=β_c_×(1-Vaccine protection) and β_c_^’*^=β_c_^’^×(1-Vaccine protection) for the scenario without and with NPIs, respectively. In each case, we used MCMC methods to estimate the final outbreak size and the total number of detectable cases during the voyage if there were 1 or 5 infectious cases boarding these ships initially.

## Results

The descriptive characteristics of COVID-19 outbreaks on each of the ships studied here are listed in Table 1. Table 2 shows the results of both parameters, transmission coefficient β (per day) and R_0_, which were estimated by the Bayesian SEIR model with and without NPIs.

**Table 1.** Characteristics of COVID-19 outbreaks from empirical data on cruise ships and warships

Name of ships	Total number of persons on board	Total number of cases (%)	Asymptomatic cases (%)	Testing strategy	NPIs	The period of NPIs	NPIs actions
*Panshi* fast combat support ship	377	44 (11.7)	17(38.6)	all sailors^a^	Yes	before 9 April	Wearing masks;
							At table in batch
							Isolated flu-like cases
						10–15 April	Onboard quarantine
						18 April	Disembarked quarantine
*Theodore*	4,779	1271^b^ (26.6)	572(43.0)	symptomatic	No	before 26 March	Isolated cases;
*Roosevelt*				cases^c^			Contacts quarantine
aircraft carrier				all crew^a^	Yes	after 27 March	Onboard quarantine;
							Disembarked quarantine
*Charles de Gaulle*	1,767	1148 (65.0)	130/1,001 (13.0)^d^	symptomatic	No	before 7 April	Isolated cases;
Aircraft Carrier				cases^e^			
				all service	Yes	after 7 April	Disembarked quarantine
				members^a^			
*Diamond Princess*	3,711	761 (20.5)	410 (53.9)		No	before 3 February	
cruise ship							
				symptomatic	Yes	After 3 February	Onboard quarantine
				cases^e^			
				All passagers^a^		After 16 February	Disembarked quarantine
*Greg Mortimer*,	217	128 (60.0)	104 (81.3)^f^	all passengers	Yes		Wearing PPE
polar expedition				and crew^a^			Onboard isolation;
cruise ship							quarantine
*Grand Princess*	3,571	122 (3.4)	Not available	symptomatic	No	before 10 March	
cruise ship				cases^a^		After 10 March	Disembarked quarantine

All passengers wearing surgical masks; The crew with N95 masks for any contact with passengers. NPIs, non-pharmaceutical interventions; PPE, personal protective equipment; R_0_, basic reproductive number; SEIR model, *susceptible-exposed-infected-recovered* model.

a Testing after disembarkation.

b Sixty-six suspected COVID-19 cases without laboratory-confirmed infection were not included.

c Testing on voyage.

d 130 asymptomatic cases were found among 1001RT-PCR confirmed cases.

e Testing during onboard quarantine.

f Pre-symptomatic plus symptomatic cases were supposed.

**Table 2.** Estimated results on parameters for COVID-19 propagation on each ship by using the Bayesian SEIR model

Name of ships	The date of disembarking or quarantine	Estimated by Bayesian SEIR model				
		β (95% CI)	σ (95% CI)	α (95% CI)	R_0_ (95% CI)	The estimated shortest time of successful containment (days)
*Panshi* fast combat support ship	15 April	0.273 (0.254–0.293) (before 9 April) 0.657(0.439–0.905) (10–15 April)	0.189 (0.184–0.194)	0.142 (0.136–0.148)	1.92 (1.80–2.06) 4.62 (3.06–6.32)	38 (16 April)
*Theodore Roosevelt* aircraft carrier	26 March to 14 April	0.919 (0.898–0.939) (before 31 March) 0.173 (0.159–0.187) (1–11 April)	0.189 (0.184–0.194)	0.143 (0.137–0.148)	6.45 (6.24–6.68) (before 31 March) 1.21 (1.12–1.30) (1–11 April)	54 (3 May)
*Charles de Gaulle* aircraft carrier	13 April	0.468 (0.444–0.488) (before 16 March) 0.733 (0.700–0.768) (17 March to 7 April) 0.240 (0.088–0.419) (8–13 April)	0.191 (0.186–0.196)	0.142 (0.137–0.149)	3.29 (3.14–3.44) (before 16 March) 5.15 (4.90–5.47) (17 March to 7 April) 1.69 (0.61–2.92) (8–13 April)	48 (15 April)
*Diamond Princess* cruise ship	16 February	0.796 (0.610–0.972)	0.187 (0.134–0.238)	0.143 (0.090–0.196)	5.73 (4.21–7.32)	28 (16 February)
*Greg Mortimer*, polar expedition cruise ship	Cases quarantine on board	0.693 (0.535–0.839)	0.189 (0.184–0.194)	0.142 (0.136–0.148)	4.87 (3.81–5.95)	22 (5 April)
*Grand Princess* cruise ship	10 March	0.735 (0.700–0.768)	0.189 (0.184–0.194)	0.142 (0.136–0.148)	5.18 (4.91–5.46)	23 (14 March)

The shortest time of successful containment measures (no member of the S class progresses to the E class): the shortest time while the number of final observed cases was within the CI of predicted total cases (E+I+R). CI, credible interval; R_0_, basic reproductive number; SEIR model, *Susceptible-Exposed-Infected-Recovered* model.

**Figure 2.**
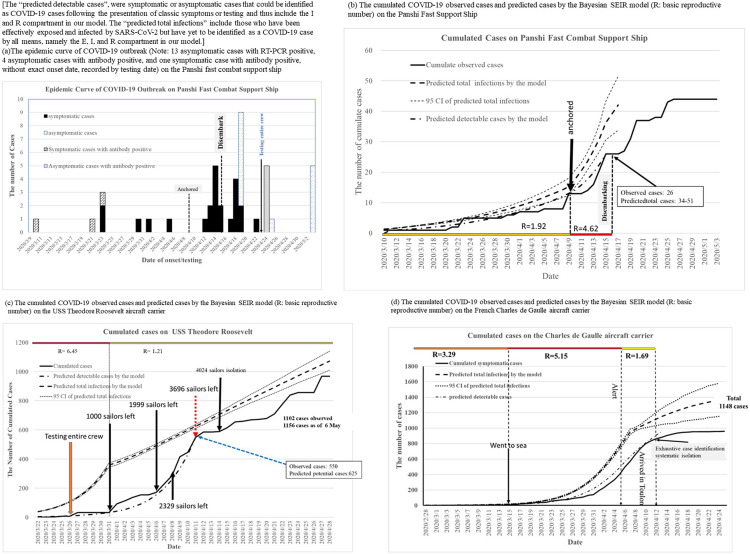
COVID-19 outbreak of warships.

**Figure 3.**
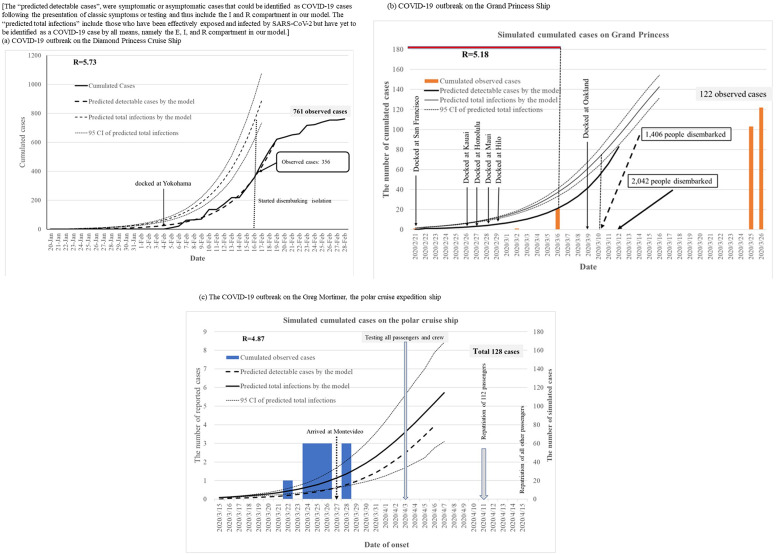
The cumulated COVID-19 observed cases and predicted cases by the Bayesian SEIR model on cruise ships. (R: basic reproductive number).

Note that the total number of COVID-19 detectable and final cases were predicted by the Bayesian SEIR model (Figure 2(b)–(d) and Figure 3; Supplementary Tables C4, C5). The reported and predicted cases by the model in these outbreaks are shown in Figures 2 and 3. The time of disembarking quarantine, such as on the *Panshi* fast combat support ship, the *Charles de Gaulle* aircraft carrier, and the *Diamond Princess* cruise ship, was highly associated with stopping the spread of COVID-19 because the potential cases in the E state and the confirmed cases (I state + R state) at that time were very close to the final size of outbreaks in our model (Table 2). In addition, the date of successful containment was later than the date of disembarking quarantine in the others. So, onboard quarantine alone did not seem sufficient in stopping the spread of COVID-19.

**Figure 4.**
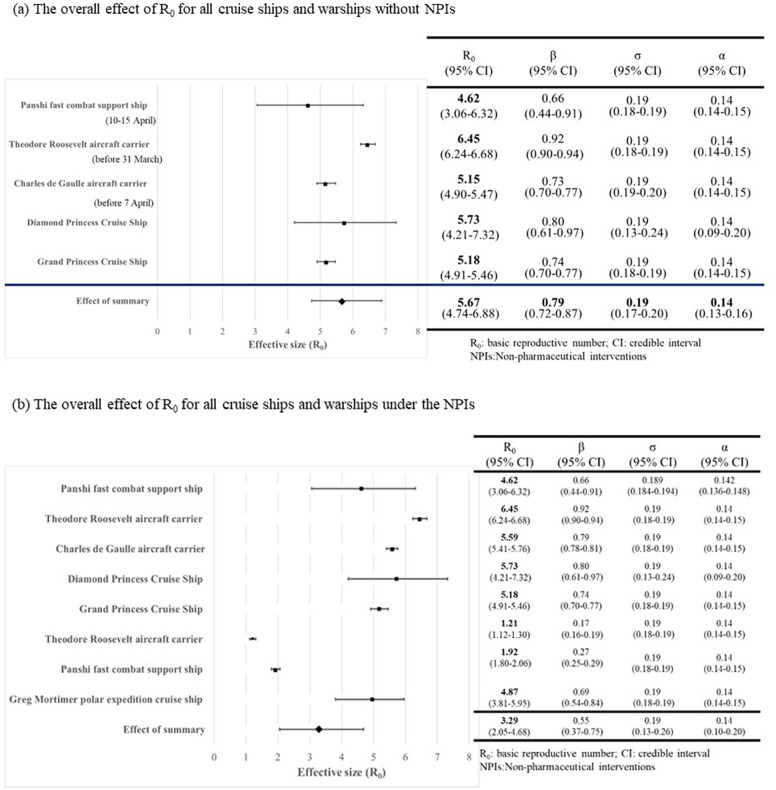
Forest plots for the overall effect of R_0_ by the Bayesian hierarchical model.

For cruise ships and warships, both estimates were relatively high with consistent figures ranging from 0.66 (95% CI:0.44-0.91) to 0.92 (95% CI:0.90-0.94) for the transmission coefficient, β (per day), and R_0_ ranging from 4.62 (95% CI:3.06-6.32) to 6.45 (95% CI:6.24-6.68), without NPIs (Figure 4). The forest plots of the overall effective size of R_0_ with or without NPIs are shown in Figure 4. The pooled estimates after meta-analysis were 0.79 (95% CI:0.72-0.87) and 5.67 (95% CI:4.74-6.88).

Transmission coefficients and effective reproductive numbers (R_t_) of COVID-19 in cruise ships and warships were reduced after the application of NPIs, now ranging from 0.17 (95% CI:0.16-0.19) to 0.69 (95% CI:0.54-0.84) for β and 1.21 (95% CI:1.12-1.30) to 4.87 (95% CI:3.81-5.95) for R_0_. The pooled estimate for R_t_ was 1.99 (95% CI:1.09-3.01), which was smaller than that of R_0_ (Figure 4b).

## Effectiveness of NPIs and vaccination

Strict infection-control measures were found to reduce COVID-19 transmission by 57.9% on the *Panshi* fast combat support ship and by 81.9% on the *Theodore Roosevelt* aircraft carrier. Meanwhile, according to the transmission coefficients estimated from the meta-analysis, the implementation of NPIs could reduce COVID-19 transmission by 49.5%.

**Figure 5.**
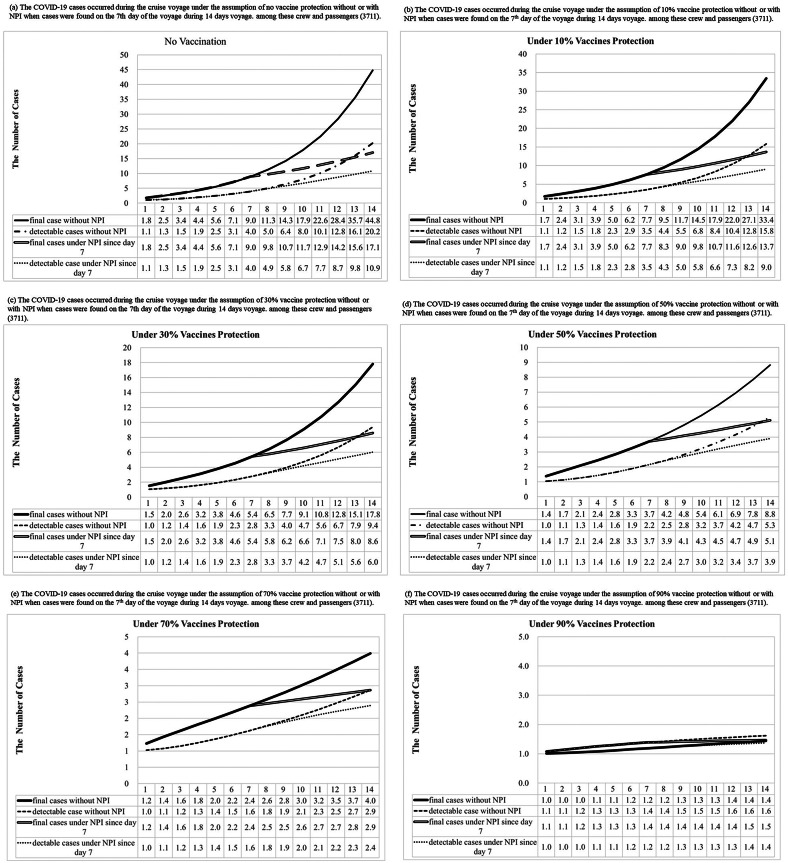
Results of COVID-19 detectable cases and predicted total cases under 0%, 10%, 30%, 50%, 70%, and 90% vaccines protection with or without NPI (non-pharmaceutical interventions) when one infectious case boarding ships initially were simulated during the voyage by the Bayesian Markov Chain Monte Carlo (MCMC) method [The “predicted observed cases”, were detectable cases that could be symptomatic or asymptomatic cases, including the I and R compartment in our model. The “total predicted cases” included detectable and undetectable infected cases that became detectable cases later. Hence, our model included them in the E, I, and R compartment.]

**Figure 6.**
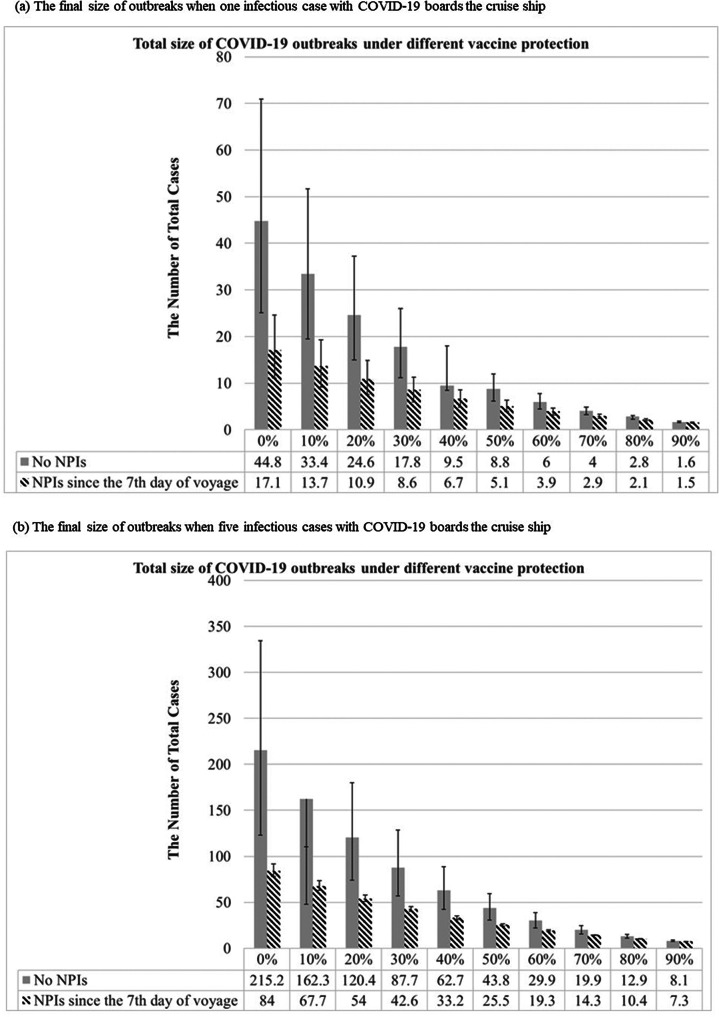
The final size of outbreaks under the different vaccine protection with or without NPIs when cases were found on the 7th day of voyage during 14 days voyage.

Figure 5 shows the number of COVID-19 detectable cases and predicted total cases under 0%, 10%, 30%, 50%, 70%, and 90% vaccine protection during the voyage of a cruise ship based on 300 simulations from the model posterior. According to the results of the Bayesian hierarchical model (Figure 4a,b), the prior distributions of σ, α, and the logarithm of R_0_ without or with the control of NPIs were assigned as gamma distribution (shape: 641.5, inverse-scale: 3409), gamma distribution (shape: 649, inverse-scale: 4557), normal distribution (mean: 1.7298, SD:0.0893), and normal distribution (mean: 0.6522, SD: 0.2453), respectively (Figure 5). In the simulation of Bayesian SEIR models with the above-mentioned parameters, there would be 45 (95% CI:25-71), 33 (95% CI:20-52), 18 (95% CI:11-26), 9 (95% CI:6-12), 4 (95% CI:3-5), and 2 (95% CI:2-2) final cases under 0%,10%, 30%, 50%, 70%, and 90% vaccine protection, respectively, without NPIs during the two weeks of the voyage (Supplementary Tables C6–C11). On a 14-day voyage where the first case is only discovered on the 7th day of the voyage, the final size of outbreaks under different vaccine protection scenarios with or without NPIs is shown in Figure 6 (Supplementary Table C12). There would be 17 (95% CI:11-25), 14 (95% CI:9-19), 9 (95% CI:6-11), 5 (95% CI:4-6), 3 (95% CI:2-3), and 2 (95% CI:1-2) final cases under 0%,10%, 30%, 50%, 70%, and 90% vaccine protection, respectively, if NPIs were implemented immediately when symptomatic cases were found on Day 7. In a scenario where all passengers and crew are under at least 70% protection from vaccination, we predicted the total size of a COVID-19 outbreak to be below 5 cases, regardless of whether NPIs are implemented or not on Day 7. The limited spread of COVID-19 (below 5 cases) can be expected under higher levels of vaccine protection even if there is more than 1 infectious case on board at the start of the voyage: for example, our simulated results show how the final size of COVID-19 cases would have been reduced to 3 (95% CI:2-3) had 70% of the passengers and crew been vaccinated compared with the corresponding 17 (95% CI:11-25) cases under the real scenario in the era without vaccination.

## Discussion

To our knowledge, this is the first comparison of COVID-19 outbreaks on different ships. The R_0_ of COVID-19 was estimated using data from a series of outbreaks on cruise ships and warships in the Bayesian SEIR model. Onboard quarantine alone did not seem sufficient to stop the spread of COVID-19. Across individual ships, we observed a reduction in COVID-19 transmission from 57.9 to 81.9% under strict infection-control measures. Subject to the assumptions of our meta-analysis, we found that R_0_ was lowered to around 73% of its original value by the introduction of NPIs on ships.

R_0_ was estimated as 5.73 for the COVID-19 outbreak on the *Diamond Princess* cruise ship in this study. This result was similar to previous reports [27–29] and consistent with other outbreaks on the *Grand Princess* and Polar expedition cruise ships in our study. We note the high transmissibility of SARS-CoV-2 on the ships in our study and the fact that it may spread via air, droplets, and fomites. Indeed, the persistence of coronaviruses, such as Severe Acute Respiratory Syndrome (SARS) coronavirus, Middle East Respiratory Syndrome (MERS) coronavirus, or endemic human coronaviruses, on inanimate surfaces for up to 9 days has been reported. [30] Diarrhea occurred in about 10% of COVID patients [31], and the presence of 2019-nCoV particles in the stool specimens indicates a fecal-oral route for coronavirus, [31, 32] which could account for why it has caused outbreaks on cruise ships with an intensity often seen in the past with gastro-causing norovirus. The fecal spread could present new challenges to the virus’s containment on cruise ships and warships. Environmental decontamination was never mentioned as a precaution taken on any of the ships studied here, and perhaps this could therefore explain why onboard quarantine and isolation alone did not appear sufficient to prevent the further spread of COVID-19 on these ships.

Nevertheless, implementing NPIs during the voyage, such as wearing masks, eating at tables in separate sittings, and isolation of flu-like cases, appeared to substantially reduce COVID-19 transmission in this study. Furthermore, the effectiveness of quarantine and isolation in reducing the number of infected passengers was 37% during the outbreak on the *Diamond Princess* cruise ship [24]. Strict NPIs, including wearing an N95 mask and full PPE, and the isolation of infected cases, could not, however, stop the transmission of COVID-19 on the *Greg Mortimer* expedition cruise ship; this may have been due to cross-contamination via the crew’s meal services and other asymptomatic cases, as the rapid antibody testing of COVID-19 patients may have resulted in a high false negative rate in the acute phase. In addition, only testing symptomatic cases and the isolation of them with their contacts was found to be insufficient, because many patients were asymptomatic [23]. Furthermore, quarantine aboard the *Panshi* fast combat support ship for a period of 6 days before disembarkation (9–15 April) seems to not have prevented the spread of COVID-19 onboard the ship.

Asymptomatic cases may be the potential sources of SARS-CoV-2 infection and the key to controlling outbreaks. In addition, asymptomatic SARS-CoV-2 transmission with high viral load has been reported [33]. Under the assumption of the same transmission probability between symptomatic and asymptomatic cases, our SEIR model fitted well to data from different outbreaks on cruise ships and warships. The fraction of pre-symptomatic transmission events out of pre-symptomatic plus symptomatic transmission events was 37% [95% confidence interval (CI), 27.5 to 45%] in a previous study [34]. High proportions of asymptomatic cases were found in outbreaks on ships, which may result from a higher proportion of latent cases. Therefore, COVID-19 continues to spread during the onboard quarantine period because these asymptomatic cases cannot be easily detected and the universal testing of all passengers and crew was rarely performed.

The cruise industry has been severely hit by the impact of COVID-19 as a major outbreak could potentially be triggered during cruise ship voyages. Even if the vaccine coverage rate was 100% among the passengers and crew, the effectiveness of a 2-dose messenger RNA (mRNA) or adenovirus-vectored COVID-19 vaccine will be imperfect and is likely to wane over time and in the face of new variants of SARS-CoV-2 [35, 36]. This study introduces some frameworks to allow the resumption of the cruise industry in the post-COVID-19 era. For example, if one asymptomatic COVID-19 person boards the cruise ship for a two-week trip and there is greater than 70% vaccine protection among the passengers and crews, then our simulation results suggest that even without NPIs there will be only limited COVID-19 cases during such a voyage. Furthermore, it appears that it may be possible to readily control the spread of COVID-19 on a cruise ship that has more than 90% vaccine protection among passengers and crew even if 5 infectious cases board the cruise ship. However, new strains of SARS-CoV2 have developed, which has led to even high vaccine coverage providing only limited protection from infection. Hence, it might seem prudent that NPIs should still be implemented immediately when any new case arising from new subvariants emerges in the post-COVID-19 pandemic era. In addition, full disembarkation and quarantine should be considered because onboard isolation and quarantine alone do not appear to be enough to stop the spread of COVID-19.

There were some limitations to our modelling and estimates of the R_0_ of COVID-19, most obviously the assumptions of a close population even with leaving the board in batches, a homogeneous random mixing population, and no difference of transmission probability between symptomatic and asymptomatic cases. The estimated results on the transmission parameters for the *Grand Princess* cruise ship may be biased towards underestimation because not everyone on board was tested [37]. In addition, the basic and effective reproductive number may be underestimated because only the final detectable cases were modelled, which is affected by the different testing strategies. All undetectable cases, such as asymptomatic cases without receiving testing, did not enter the infectious state in this study. However, consistent results were obtained from the different outbreaks under consideration. Most of the outbreaks that took place on cruise ships and warships were reported during the Wuhan strain and the Alpha VOC period. Furthermore, only the aggregate data on the number of cases that evolved through the clustered event without detailed information on the history of previous infections were available. The evaluation of the protective effectiveness derived from the infection by ancestral strains was thus hampered. In addition, potential changes in the reproduction number of SARS-CoV-2 variants [38] are not considered in our model. However, as the omicron variants and subvariants had higher transmissibility and immune escape than the ancestral strain [39], and vaccination protection against emerging variant strains may not be desirable due to weaning immunity. The effectiveness of the vaccine against omicron infections may be reduced compared to its effectiveness against the previous strains. Hence, the results of our simulation will be the best scenarios for predicting the future challenges in the post-COVID-19 pandemic era. However, given a body of evidence indicating an enhanced and long-lasting immune response built on hybrid immunity [40], passengers and crew who have recovered from previous infections with updated vaccination status can further secure the safety on board in terms of the risk of the COVID-19 outbreak.

In conclusion, we find a limited spread of SARS-CoV-2 during the two weeks of a voyage under at least over 70% of vaccine protection with NPIs. Usage of masks by the crew for any contact with passengers is advisable. Furthermore, testing of symptomatic cases and their contacts, disembarkation quarantine, and the isolation of symptomatic/ asymptomatic cases should nevertheless be performed as soon as possible when COVID-19 cases are found on ships, because of the high proportion of asymptomatic/pre-symptomatic cases likely to be present. Of course, a higher rate of updated vaccine protection among the crew and passengers is also necessary for stopping the spread of COVID-19 on cruise ships.

## Data Availability

The data that support the findings of this study are openly available in the public domain and in the literature [15–25]. They are listed in the Supplementary material.

## Supplementary material

For supplementary material accompanying this paper visit https://doi.org/10.1017/S0950268823000821. click here to view supplementary material
