# Supplementary material for: Can ship travel contain COVID-19 outbreak after re-opening: a Bayesian meta-analysis
Source: Epidemiol Infect. 2023 May 25;151:e99. doi: 10.1017/S0950268823000821 (PMC10317826; doi:10.1017/S0950268823000821)
Supplement: Supplementary file 1 [file S0950268823000821sup001.docx]

**Supplementary material**

**Can Ship Travel Contain COVID-19 Outbreak After Re-Opening: A Bayesian Meta-analysis**

1. **Data sources**

The data on COVID-19 outbreaks on cruise ships and warships were obtained from public messages and literature [1], which were used to estimate the parameters of the compartmental model. A line list regarding the data reported for each ship were detailed in **Table A1** for warships (**Table A1 (a)** to **(c)**) and cruise ships (**Table A1 (d)** to **(f)**) with the corresponding epidemic curve presented in **Figure C1** and **C2** (warships) and **Figure C3** (cruise ships). The context for each outbreak on board was briefed in the following **Appendix A.1** (warship) and **A.2** (cruise ship).

**A.1 COVID-19 outbreaks on the warship**

***Taiwan Panshi fast combat support ship***

The Panshi fast combat support ship departed Kaohsiung of Taiwan with 377 sailors on 5 March 2020 and docked in Palau from 12-15 March 2020. All sailors had to wear face masks, and they had to be at table in batches during this mission. In addition, all flu-like cases were isolated on board on the voyage. It anchored in Kaohsiung on 9 April 2020, and all sailors remained on board from then until disembarkation on 15 April 2020. Unfortunately, three suspected COVID-19 cases were reported because they had COVID-19 associated symptoms on 17 April. Hence, all sailors were isolated or quarantined on 18 April. A total of 36 COVID-19 cases, including 32 men and four women, ranged between 20 and 50 years of age on the Panshi fast combat support ship were confirmed to have the coronavirus, including 13 asymptomatic cases. All sailors were quarantined for 14 days since April 18 and tested for COVID-19 antibody on 23 April; another eight sailors had positive antibody testing, of which 4 were asymptomatic. In total, 44 COVID-19 cases were confirmed and the epidemic curve is shown in **Figure A1(a)**. The onset date of index case was 10 March. Therefore, the date of the first infectious case was assumed on 10 March in the model.

***USS Theodore Roosevelt aircraft carrier***

Theodore Roosevelt aircraft carrier departed Naval Base San Diego with 4,865 sailors and it arrived in Da-Nang, Vietnam on 5 March 2020. It departed Da-Nang on 9 March (the date of the first infectious case was assumed in the model). The first COVID-19 case was confirmed on 15 March [2, 3]. It then docked in Guam and the entire crew was tested for COVID-19 on, 26 March with sailors gradually leaving the ship: 1,000 sailors left the ship on 31 March, with a further 999 leaving on the 6^th^ April (1999 disembarkations in total), 230 leaving on the 8^th^ April (2329 total disembarkations), 1367 leaving on the 11^th^ April (3696 total disembarkations) and 328 leaving on the 14^th^ April (4024 total disembarkations). 1102 COVID-19 infections were reported by 30^th^ April. With a final count of 1271 confirmed infections reported on 18 May [4].

***French Charles de Gaulle***

During 4-month mission, French Charles de Gaulle aircraft carrier was docked in Limassol (Cyprus, 21–26 February) and Brest (Western France, 13–16 March) and all sailors had no contact with the outside world since it went to sea after 16 March 2020. [4] An outbreak was reported on 7 April. Hence, the aircraft carrier was ordered back to base in Toulon where it arrived on 13 April. Exhaustive case identification with systematic isolation was performed after arrived in Toulon. After the outbreak investigation, the onset date of the first confirmed case was on 28 February. Finally, 1,148 COVID-19 cases among 1,767 members of this aircraft carrier were reported [4].

**A.2 COVID-19 outbreak on cruise ships**

***Diamond Princess Cruise Ship***

Diamond Princess cruise ship departed from the Port of Yokohama on January 20 for a round-trip of Southeast Asia with 2666 passengers and 1045 crew on board. The first confirmed COVID-19 case was reported on 1 February and the onset date of the index case was on January 19, 2020. Therefore, the date of the first infectious case was on January 20 in the model. The ship docked at the port of Keelung, Taiwan on 31 January where smart digital contact tracing and automated alert message for self-restriction were performed to prevent the spread of COVID-19 in Taiwan [5]. The cruise ship had been quarantined since arriving in Yokahama on 3 February. On-site quarantine for crews and passengers and landing isolation for cases were performed. Disembarkation and subsequent quarantine for all persons started on 16 February. The earliest and most confirmed crew members with COVID-19 worked in food service workers [6]. Seven hundred and sixty-one confirmed cases with COVID-19, including the index case, cases confirmed in Japan and outside Japan, had been reported on 29 February [7].

***Grand Princess Cruise Ship***

The Grand Princess cruise ship departed from San Francisco on 21 February, 2020 for a round-trip, from San Francisco, California, making four stops in Mexico with 2460 passengers and 1111 crew on board [8]. The first confirmed COVID-19 case, returning from a Mexican on Grand Princess between 11- 21 February, was reported on 21 February, the date of the first infectious case in the model, and died on 4 March. The ship was ordered to return to San Francisco. In addition, on board quarantine was ordered on 4 March. Twenty-one cases were confirmed on 6 March. The ship docked in Oakland and 1,406 people had disembarking quarantine on 10 March. By 12 March. 2,042 people had disembarked. One hundred and twenty-two confirmed cases with COVID-19 had been reported on 26 March [9]. Not all passengers were tested for COVID-19, even though some ones had symptoms upon disembarkation [10].

***Greg Mortimer: Polar expedition cruise ship***

The polar expedition cruise ship, Greg Mortimer cruise ship, departed from Ushuaia, Argentina on 15 March for a planned 21-day cruise of the Antarctic Peninsula, including Falkland Islands, South Georgia and Drake Passage, with 217 passengers and crew on board. The onset date of first confirmed case was 22 March, but the first infectious case was assumed on 15 March in our model. On-board isolation and quarantine were immediately done and surgical masks issued to all. The crew still performed duties, including meal services to the cabin doors, with N95 masks for any contact with passengers but rooms were not serviced. For any contact with any febrile patients, full personal protective equipment was used [11]. It arrived at Montevideo, Uruguay on 27 March, and was permitted to dock on 10 April. Of 10 COVID-19 cases, there were 4 crew and 6 passengers as of 26 March. Testing all passengers and crew for COVID-19 was performed on 3 April. One hundred and twenty-eight confirmed cases with COVID-19 had been reported. Estimated data was obtained from the simulation of a deterministic SEIR model from 15 March to 17 April because there were no enough data for estimation during this period.

**Table A1. The number of cumulated reported COVID-19 cases on warships and cruise ships.**

**(a) The Panshi fast combat support ship (N=377)**

| **The Panshi fast combat support ship (N=377)** | | | |
| --- | --- | --- | --- |
| **Day of Voyage** | **Date** | **Cumulated reported cases** | **Notes** |
| 1 | 2020/3/10 | 1 | The onset date of the index cases.  The date of the first infectious case was assumed in the model. |
| 2 | 2020/3/11 | 1 |  |
| 3 | 2020/3/12 | 1 |  |
| 4 | 2020/3/13 | 1 |  |
| 5 | 2020/3/14 | 1 |  |
| 6 | 2020/3/15 | 1 |  |
| 7 | 2020/3/16 | 1 |  |
| 8 | 2020/3/17 | 1 |  |
| 9 | 2020/3/18 | 1 |  |
| 10 | 2020/3/19 | 1 |  |
| 11 | 2020/3/20 | 1 |  |
| 12 | 2020/3/21 | 2 |  |
| 13 | 2020/3/22 | 2 |  |
| 14 | 2020/3/23 | 5 |  |
| 15 | 2020/3/24 | 5 |  |
| 16 | 2020/3/25 | 5 |  |
| 17 | 2020/3/26 | 5 |  |
| 18 | 2020/3/27 | 5 |  |
| 19 | 2020/3/28 | 5 |  |
| 20 | 2020/3/29 | 5 |  |
| 21 | 2020/3/30 | 6 |  |
| 22 | 2020/3/31 | 6 |  |
| 23 | 2020/4/1 | 7 |  |
| 24 | 2020/4/2 | 7 |  |
| 25 | 2020/4/3 | 7 |  |
| 26 | 2020/4/4 | 7 |  |
| 27 | 2020/4/5 | 8 |  |
| 28 | 2020/4/6 | 8 |  |
| 29 | 2020/4/7 | 8 |  |
| 30 | 2020/4/8 | 8 |  |
| 31 | 2020/4/9 | 13* | Anchored in Kaohsiung |
| 32 | 2020/4/10 | 13 |  |
| 33 | 2020/4/11 | 13 |  |
| 34 | 2020/4/12 | 14 |  |
| 35 | 2020/4/13 | 16 |  |
| 36 | 2020/4/14 | 21 |  |
| 37 | 2020/4/15 | 26* |  |
| 38 | 2020/4/16 |  |  |
| 39 | 2020/4/17 |  |  |
| 40 | 2020/4/18 | 44 | All sailors were isolated or quarantined |

**(b) The USS Theodore Roosevelt aircraft carrier**

| **The USS Theodore Roosevelt aircraft carrier (N=4779)** | | | |
| --- | --- | --- | --- |
| **Day** | **Date** | **Cumulated reported cases** | **Notes** |
| 1 | 2020/3/15 | 1 | The date of first confirmed cases.  (the date of the first infectious case was assumed in the model) |
| 2 | 2020/3/16 | 1 |  |
| 3 | 2020/3/17 | 1 |  |
| 4 | 2020/3/18 | 1 |  |
| 5 | 2020/3/19 | 1 |  |
| 6 | 2020/3/20 | 1 |  |
| 7 | 2020/3/21 | 1 |  |
| 8 | 2020/3/22 | 1 |  |
| 9 | 2020/3/23 | 1 |  |
| 10 | 2020/3/24 | 3 |  |
| 11 | 2020/3/25 | 8 |  |
| 12 | 2020/3/26 | 8 | Testing entire crew |
| 13 | 2020/3/27 | 33 |  |
| 14 | 2020/3/28 | 33 |  |
| 15 | 2020/3/29 | 33 |  |
| 16 | 2020/3/30 | 33 |  |
| 17 | 2020/3/31 | 33 | 1000 sailors left |
| 18 | 2020/4/1 | 93 |  |
| 19 | 2020/4/2 | 114 |  |
| 20 | 2020/4/3 | 137 |  |
| 21 | 2020/4/4 | 155 |  |
| 22 | 2020/4/5 | 155 |  |
| 23 | 2020/4/6 | 172 | 1999 sailors left |
| 24 | 2020/4/7 | 230 |  |
| 25 | 2020/4/8 | 286 |  |
| 26 | 2020/4/9 | 416 |  |
| 27 | 2020/4/10 | 447 |  |
| 28 | 2020/4/11 | 550 | 3696 sailors left |
| 29 | 2020/4/12 | 585 |  |
| 30 | 2020/4/13 | 585 |  |
| 31 | 2020/4/14 | 589 | 4024 sailors isolated |
| 32 | 2020/4/15 | 615 |  |
| 33 | 2020/4/16 | 655 |  |
| 34 | 2020/4/17 | 660 |  |
| 35 | 2020/4/18 | 669 |  |
| 36 | 2020/4/19 | 672 |  |
| 37 | 2020/4/20 | 678 |  |
| 38 | 2020/4/21 | 710 |  |
| 39 | 2020/4/22 | 777 |  |
| 40 | 2020/4/23 | 840 |  |
| 41 | 2020/4/24 | 856 |  |
| 42 | 2020/4/25 | 856 |  |
| 43 | 2020/4/26 | 856 |  |
| 44 | 2020/4/27 | 969 |  |
| 45 | 2020/4/28 | 969 |  |
| 46 | 2020/4/29 | 1102 |  |

**(c) The French Charles de Gaulle aircraft carrier**

|  | | | **The French Charles de Gaulle aircraft carrier (N=1767)** | | |
| --- | --- | --- | --- | --- | --- |
| **Day** | **Date** | **symptomatic cases** | | **Cumulated symptomatic cases** | **Notes** |
| 1 | 2020/2/28 | 1 | | 1 | The onset date of the first symptomatic case. |
| 2 | 2020/2/29 | 0 | | 1 |  |
| 3 | 2020/3/1 | 0 | | 1 |  |
| 4 | 2020/3/2 | 1 | | 2 |  |
| 5 | 2020/3/3 | 0 | | 2 |  |
| 6 | 2020/3/4 | 0 | | 2 |  |
| 7 | 2020/3/5 | 1 | | 3 |  |
| 8 | 2020/3/6 | 0 | | 3 |  |
| 9 | 2020/3/7 | 0 | | 3 |  |
| 10 | 2020/3/8 | 0 | | 3 |  |
| 11 | 2020/3/9 | 0 | | 3 |  |
| 12 | 2020/3/10 | 1 | | 4 |  |
| 13 | 2020/3/11 | 1 | | 5 |  |
| 14 | 2020/3/12 | 0 | | 5 |  |
| 15 | 2020/3/13 | 0 | | 5 | Charles de Gaulle was docked in Brest from 13 to 16 March. |
| 16 | 2020/3/14 | 0 | | 5 |  |
| 17 | 2020/3/15 | 5 | | 10 |  |
| 18 | 2020/3/16 | 1 | | 11 |  |
| 19 | 2020/3/17 | 9 | | 20 |  |
| 20 | 2020/3/18 | 2 | | 22 |  |
| 21 | 2020/3/19 | 5 | | 27 |  |
| 22 | 2020/3/20 | 11 | | 38 |  |
| 23 | 2020/3/21 | 0 | | 38 |  |
| 24 | 2020/3/22 | 2 | | 40 |  |
| 25 | 2020/3/23 | 19 | | 59 |  |
| 26 | 2020/3/24 | 4 | | 63 |  |
| 27 | 2020/3/25 | 11 | | 74 |  |
| 28 | 2020/3/26 | 1 | | 75 |  |
| 29 | 2020/3/27 | 14 | | 89 |  |
| 30 | 2020/3/28 | 9 | | 98 |  |
| 31 | 2020/3/29 | 11 | | 109 |  |
| 32 | 2020/3/30 | 23 | | 132 |  |
| 33 | 2020/3/31 | 9 | | 141 |  |
| 34 | 2020/4/1 | 49 | | 190 |  |
| 35 | 2020/4/2 | 40 | | 230 |  |
| 36 | 2020/4/3 | 54 | | 284 |  |
| 37 | 2020/4/4 | 50 | | 334 |  |
| 38 | 2020/4/5 | 70 | | 404 |  |
| 39 | 2020/4/6 | 93 | | 497 |  |
| 40 | 2020/4/7 | 76 | | 573 | An outbreak was reported |
| 41 | 2020/4/8 | 88 | | 661 |  |
| 42 | 2020/4/9 | 76 | | 737 |  |
| 43 | 2020/4/10 | 63 | | 800 |  |
| 44 | 2020/4/11 | 37 | | 837 |  |
| 45 | 2020/4/12 | 26 | | 863 |  |
| 46 | 2020/4/13 | 19 | | 882 | Arrived in Toulon and exhaustive case identification with systematic isolation |
| 47 | 2020/4/14 | 24 | | 906 |  |
| 48 | 2020/4/15 | 16 | | 922 |  |
| 49 | 2020/4/16 | 15 | | 937 |  |
| 50 | 2020/4/17 | 9 | | 946 |  |
| 51 | 2020/4/18 | 4 | | 950 |  |
| 52 | 2020/4/19 | 4 | | 954 |  |
| 53 | 2020/4/20 | 1 | | 955 |  |
| 54 | 2020/4/21 | 0 | | 955 |  |
| 55 | 2020/4/22 | 0 | | 955 |  |
| 56 | 2020/4/23 | 1 | | 956 |  |
| 57 | 2020/4/24 | 1 | | 957 |  |
| 58 | 2020/4/25 | 2 | | 959 | 1148 confirmed cases among 1767 members |

**(d) The Diamond Princess cruise ship (N=3711)**

| **The Diamond Princess cruise ship (N=3711)** | | | |
| --- | --- | --- | --- |
| **Day** | **Date** | **Cumulated reported cases** | **Notes** |
| 1 | 2020/1/20 | 1 | The index case boarded and the onset date was 19 January.  The date of the first infectious case in the model. |
| 2 | 2020/1/21 | 1 |  |
| 3 | 2020/1/22 | 1 |  |
| 4 | 2020/1/23 | 1 |  |
| 5 | 2020/1/24 | 1 |  |
| 6 | 2020/1/25 | 1 |  |
| 7 | 2020/1/26 | 1 |  |
| 8 | 2020/1/27 | 1 |  |
| 9 | 2020/1/28 | 1 |  |
| 10 | 2020/1/29 | 1 |  |
| 11 | 2020/1/30 | 1 |  |
| 12 | 2020/1/31 | 1 |  |
| 13 | 2020/2/1 | 1 |  |
| 14 | 2020/2/2 | 1 |  |
| 15 | 2020/2/3 | 1 | On-board quarantine since arriving in Yokahama |
| 16 | 2020/2/4 | 1 |  |
| 17 | 2020/2/5 | 11 |  |
| 18 | 2020/2/6 | 21 |  |
| 19 | 2020/2/7 | 62 |  |
| 20 | 2020/2/8 | 65 |  |
| 21 | 2020/2/9 | 71 |  |
| 22 | 2020/2/10 | 136 |  |
| 23 | 2020/2/11 | 136 |  |
| 24 | 2020/2/12 | 175 |  |
| 25 | 2020/2/13 | 219 |  |
| 26 | 2020/2/14 | 219 |  |
| 27 | 2020/2/15 | 286 |  |
| 28 | 2020/2/16 | 356 | Started disembarking isolation |
| 29 | 2020/2/17 | 455 |  |
| 30 | 2020/2/18 | 557 |  |
| 31 | 2020/2/19 | 622 |  |
| 32 | 2020/2/20 | 635 |  |
| 33 | 2020/2/21 | 651 |  |
| 34 | 2020/2/22 | 659 |  |
| 35 | 2020/2/23 | 718 |  |
| 36 | 2020/2/24 | 722 |  |
| 37 | 2020/2/25 | 740 |  |
| 38 | 2020/2/26 | 706 |  |
| 39 | 2020/2/27 | 706 |  |
| 40 | 2020/2/28 | 761 |  |

**(e) The Grand Princess cruise ship**

| **The Grand Princess cruise ship (N=3571)** | | | |
| --- | --- | --- | --- |
| **Day** | **Date** | **Cumulated reported cases** | **Notes** |
| 1 | 2020/2/21 |  | The reported date of the first confirmed case  The date of the first infectious case in the model. |
| 2 | 2020/2/22 |  |  |
| 3 | 2020/2/23 |  |  |
| 4 | 2020/2/24 |  |  |
| 5 | 2020/2/25 |  |  |
| 6 | 2020/2/26 |  | Docked at Kauai |
| 7 | 2020/2/27 |  | Docked at Honolulu |
| 8 | 2020/2/28 |  | Docked at Maui |
| 9 | 2020/2/29 |  |  |
| 10 | 2020/3/1 |  |  |
| 11 | 2020/3/2 | 1 |  |
| 12 | 2020/3/3 |  |  |
| 13 | 2020/3/4 |  |  |
| 14 | 2020/3/5 |  |  |
| 15 | 2020/3/6 | 21 |  |
| 16 | 2020/3/7 |  |  |
| 17 | 2020/3/8 |  |  |
| 18 | 2020/3/9 |  | Docked at Oakland |
| 19 | 2020/3/10 |  | 1,406 people had disembarking quarantine |
| 20 | 2020/3/11 |  |  |
| 21 | 2020/3/12 |  | 2,042 people had disembarking quarantine |
| 22 | 2020/3/13 |  |  |
| 23 | 2020/3/14 |  |  |
| 24 | 2020/3/15 |  |  |
| 25 | 2020/3/16 |  |  |
| 26 | 2020/3/17 |  |  |
| 27 | 2020/3/18 |  |  |
| 28 | 2020/3/19 |  |  |
| 29 | 2020/3/20 |  |  |
| 30 | 2020/3/21 |  |  |
| 31 | 2020/3/22 |  |  |
| 32 | 2020/3/23 |  |  |
| 33 | 2020/3/24 |  |  |
| 34 | 2020/3/25 | 103 |  |
| 35 | 2020/3/26 | 122 |  |

**(f) The Greg Mortimer polar expedition cruise ship (N=217)**

| **The Greg Mortimer polar expedition cruise ship (N=217)** | | | |
| --- | --- | --- | --- |
| **Day** | **Date** | **Cumulated reported cases** | **Notes** |
| 1 | 2020/3/15 |  | The date of the first infectious case in the model. |
| 2 | 2020/3/16 |  |  |
| 3 | 2020/3/17 |  |  |
| 4 | 2020/3/18 |  |  |
| 5 | 2020/3/19 |  |  |
| 6 | 2020/3/20 |  |  |
| 7 | 2020/3/21 |  |  |
| 8 | 2020/3/22 | 1 | The onset date of first confirmed case |
| 9 | 2020/3/23 | 1 |  |
| 10 | 2020/3/24 | 4 |  |
| 11 | 2020/3/25 | 7 |  |
| 12 | 2020/3/26 | 10 |  |
| 13 | 2020/3/27 | 10 |  |
| 14 | 2020/3/28 | 13 |  |
| 15 | 2020/3/29 | 13 |  |
| 16 | 2020/3/30 | 13 |  |
| 17 | 2020/3/31 | 13 |  |
| 18 | 2020/4/1 | 13 |  |
| 19 | 2020/4/2 | 13 |  |
| 20 | 2020/4/3 | 21 | Testing all passengers was started |
| 21 | 2020/4/4 | 22 |  |
| 22 | 2020/4/5 | 25 |  |
| 23 | 2020/4/6 | 26 |  |
| 24 | 2020/4/7 | 27 |  |
|  |  |  | Finally,128 confirmed cases were found. |

**B. The deterministic and Bayesian compartment (SEIR) models for the transmission of COVID-19 on ships.**

The parameters of COVID-19 outbreaks on ships were estimated with the Bayesian SEIR model after dealing with missing data which was obtained from the deterministic SEIR model. The software of MATLAB version 9.30.713579 (MathWorks, USA) was used for the deterministic model. The Markov chain Monte Carlo (MCMC) procedure in the SAS software (release 9.4) was used to estimate the transmission parameters and R_0_ regarding COVID-19 transmission on board.

**B.1 The deterministic SEIR model**

A deterministic Susceptible-Exposed-Infected-Recovered (SEIR) model was used for imputation of missing data in the outbreaks on ships, such as Charles de Gaulle aircraft carrier, Grand Princess Cruise Ship, and polar expedition cruise ship. The four compartments of COVID-19 infection, SEIR, correspond to Susceptible (S), exposed to cases (E), infectious cases (I), and cases with isolation or immunity after recovery (R). **Figure B1 (a)** shows the deterministic SEIR model depicting the transmission of COVID-19 through the four status of infection specified as above.

A nonlinear ordinary differential equations of the COVID-19 model were used to describe the dynamic changes of each state as follows.

$\frac{dS\text{(t)}}{dt}=-\beta\frac{\text{S(t)}}{\text{N}}\text{I(t)}$ (1)

$\frac{d\text{E(t)}}{dt}=\beta\frac{\text{S(t)}}{\text{N}}\text{I(t)-}\sigma\text{E(t)}$ (2)

$\frac{d\text{I(t)}}{dt}=\sigma\text{E(t)-}\alpha\text{I(t)}$ (3)

$\frac{d\text{R(t)}}{dt}=\alpha\text{I(t)}$ , (4)

where β denotes the transmission coefficient, overall transmissibility with contact rate; σ denotes reciprocal of the incubation period (5 days) [12] and it was set to be 0.2; α denotes reciprocal of the infectious period or time to removal (7 days) and it was set to be 0.1428. The formula of deriving the basic reproductive number (R_0_) is $\frac{\beta}{\alpha}$. We assumed a constant duration of the infectious period for the outbreaks on cruise ships and warships due the outbreaks caused by similar strain. By fitting the COVID-19 cases simulated from the deterministic SEIR model with the observed ones, the estimated results on the transmission coefficient (β), basic reproductive number (R_0_), and the time of successful containment for COVID-19 outbreaks on the ships can be derived.

Overall transmissibility determined mainly by contact rate is highly associated with control measures such as cases isolation, policy of social distancing and quarantine, and the regulation on wearing face masks on board. The impact of these containment measures was evaluated by comparing the estimated results on the transmission coefficient (β) and corresponding reproduction number during the periods of outbreak on board for each ship.

Some common assumptions in our deterministic SEIR model are as follows. (1) Homogeneous random mixing population, including crew and passengers; (2) fixed incubation and fixed infectious period; (3) no difference of transmission probability between symptomatic and asymptomatic cases; (4) the days between reporting date and onset date are similar in the same outbreak; (5) all ones in exposed state became cases (I).

Propagation of COVID-19 outbreak on cruise ships and warships and the impact of control measures for the spreading were evaluated via transmission coefficient which in was captured by the transmissibility with the contact rate of SEIR model. Hence, the moment of successful containment measures, namely no susceptible persons (S) progress to the exposed state (E), occurred when the total number of potential cases in the exposed compartment (E) and confirmed COVID-19 cases (I and R compartments) was equal to the total number of the final observed confirmed COVID-19 cases on each ship. The simulation of the deterministic SEIR model was performed by using the software of MATLAB version 9.30.713579 (MathWorks, USA).

**B.2 The Bayesian SEIR model and the Bayesian meta-analysis model**

Extended from the compartments defined in **Appendix B.1** with a Bayesian SEIR model, the propagation and transmission of COVID-19 on each ship can be modelled (**Figure B1(b)**) and the pooled estimate across ships (**Figure 1**) can be also modelled by using the random-effect. **Figure B1 (b)** shows how to apply the directed acyclic graphic (DAG) diagram to model the relationship between empirical data and the estimated parameters. We used a Poisson distribution (Y_IR, t_ ~ Poisson (μ_It_+μ_Rt_)) or a Binomial distribution (Y_IR, t_ ~ Binomial (N, *P*_IR, t_), *P*_IR, t_ = $\frac{\mu\mathrm{It}+\mu\mathrm{Rt}}{N}$) for modelling the observed number of COVID-19 case on board at time t (Y_IR_, _t_) and a normal distribution (Y_SE, t_ ~ Normal (μ_St_+μ_Et_ , 10^4^)) for modelling the number of susceptible and exposed, the complement of COVID-19 cases (Y_IR, t_ = N- Y_SE, t_), at time *t*. Following the notation defined in the deterministic SEIR model, β, α, and σ represents the transmission coefficient, the recovery rate or the reciprocal of time to removal, and the reciprocal of the incubation period on ship. Note that the prior distributions of the parameters (β, α, and σ) were also specified in the Bayesian SEIR model of Figure B1(b). A noninformative prior of Gamma (0.001, 0.0001) was elicited for the parameter of transmission coefficient (β). Regarding the two parameters of disease evolution, namely the inverse of the incubation period (σ) and the recovery rate (α), informative priors were used. The outbreaks on the ships that were considered in our study all occurred during the early period of COVID-19 pandemic before Jun 2020, by when the D614G were the dominant VOC. The results of previous studies conducted during the early period on the two clinical parameters were thus borrowed as the prior information. Specifically, the average incubation period of 5.25 days and the average infectious period of 7 days was used. [7, 12-15] To take into account the uncertainty inherited within the reported results on the two clinical parameters, the range of 4 to 7 days for the incubation period and 5 to12 days for the infectious period were applied. Translated from the results derived from this clinical observation, the information prior of σ ~ Gamma (53, 278) and α ~ Gamma (24, 168) were thus elicited for the inverse of incubation period (σ) and the recovery rate (α). (**Figure B1(b)**)

The observed number of daily COVID-19 cases in each ship thus provides sufficient information on the parameters of Poisson distribution or Binomial distribution and Normal distribution specified above, which in turn were determined by the parameters encoded in the Bayesian SEIR model through the ordinary differential equations (1) to (4) [8].

Regarding the Bayesian SEIR model of meta-analysis for COVID-19 propagation on ships (**Figure 1** in the main text), which is an extension of the Bayesian SEIR model of **Figure B1 (b)**, a log-linear random effect model was used for the derivation of the results of meta-analysis. We assume that the impact of NPIs is the same on warships and cruise ships Specifically, the derivation for the meta-analysis of the three parameters were elaborated as follows. Taking the transmission coefficient, β, as an illustration, the log-linear random effect model can be specified by

log(β_k_)= a*_k_*+r*_k_*

r*_k_* ~(0, τ_β_)

a*_k_* ~ Normal (a_c_, *v*_c_), (5)

where *k* represents each ship enrolled in the study, r*_k_* is the variance of the posterior distribution of log-transformed transmission coefficient for the *k*^th^ ship. The results of common transmission coefficient without NPIs, β_c_, can thus be derived by exp(a_c_). Meta-analysis for the common value of the reciprocal of incubation period (σ*_k_*) and recovery rate (α_c_) were derived similarly. Regarding the meta-analysis for types of ships including that of warship, cruise ship, and that with and without NPIs, the log-linear random-effect model was applied to the corresponding types of ships.

The inferences on the parameters were drawn on the basis of the mean values and the 95% highest posterior density (HPD) derived from 150000 times of sampling simulation with the burn-in interval of 5000 times and the thinning interval of 50.

**Figure B1. The SEIR compartment model for COVID-19 transmission on board.**

1. **Deterministic SEIR model**


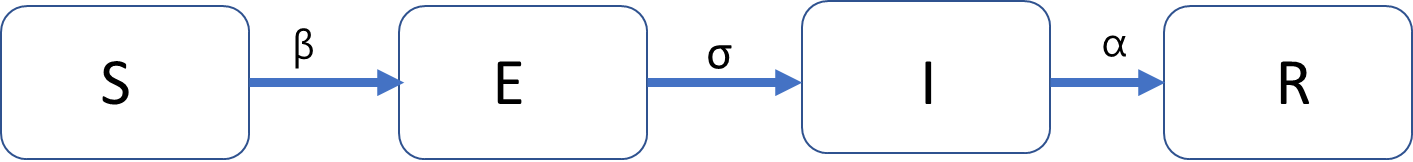


1. **Bayesian DAG of the SEIR model**

**
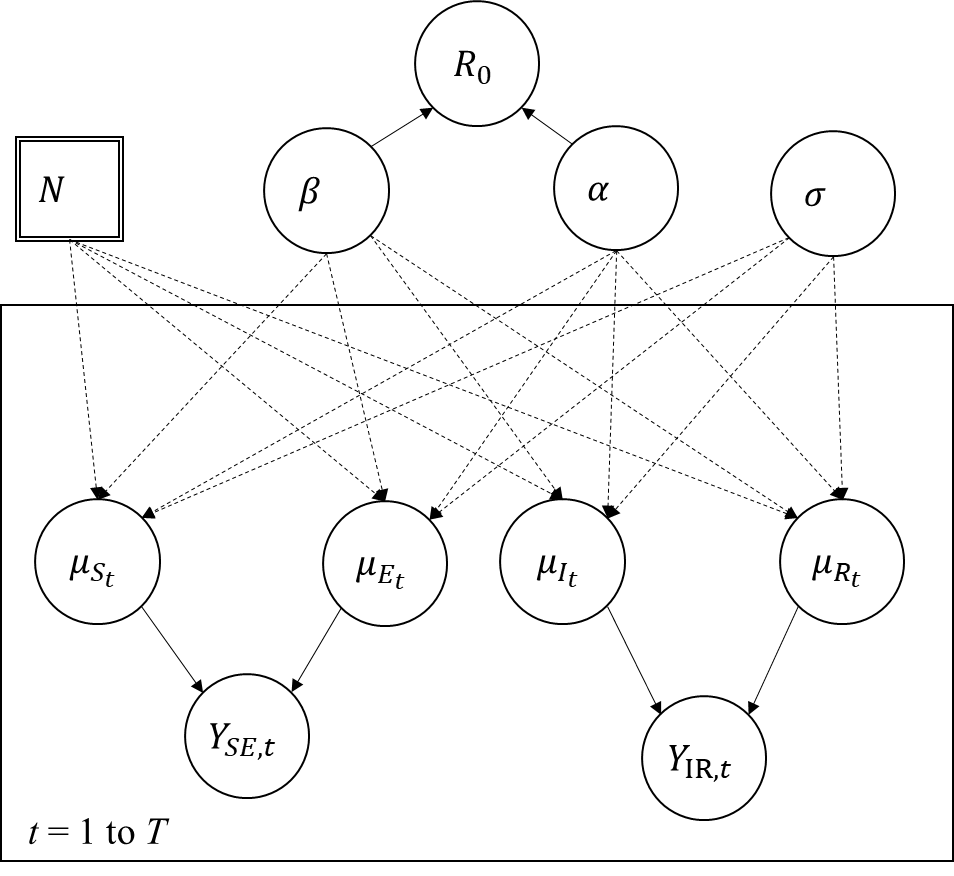
**

N: Total number of subjects on board

T: Total duration of COVID-19 outbreak on the ship

Y_IR, t_: Observed number of COVID-19 cases at time *t*

Y_SE,t_ = N-Y_IR, t_

μ_St_, μ_Et_, μ_It_, μ_Rt_ : Average number of subjects in compartments S, E, I, and R at

time t, respectively.

R_0_ = $\frac{\beta}{\alpha}$

*Three prior distributions of the parameters are specified as follows*

β ~ Gamma (0.001, 0.001)

α ~ Gamma (24, 168)

σ ~ Gamma (53, 278)

**C. Estimated results derived by using the deterministic and Bayesian SEIR model.**

The estimated results on the transmission coefficient (β) and basic reproductive number (R_0_) for each ship derived by using deterministic SEIR model are listed in **Table C1** with the number of COVID-19 cases regarding the compartment I+R and compartment E driven by the force of COVID-19 transmission, β, listed in **Table C2** (warships) and **Table C3** (cruise ships). The fitted results compared to the observed number of COVID-19 cases are presented in **Figure C1(b)** and **Figure C2** (warships) and **Figure C3** (cruise ships)

The results of predicted total potential COVID-19 cases by Bayesian SEIR model are listed in **Table C4** (warships) and **Table C5** (cruise ships). In the Bayesian SEIR model, the expected number of the component I and R were modeled by using a Binomial distribution for the outbreak of Charles de Gaulle aircraft carrier, and by using a Poisson distribution for the others on the basis of the convergence status of posterior sampling history determined by the trace plot of MCMC procedure.

The Results of COVID-19 detectable cases and predicted total cases under 0%, 10%, 30%, 50%, 70% and 90% vaccines protection with or without NPIs (non-pharmaceutical interventions) when one infectious case boarding ships initially were simulated during the voyage by the Bayesian Markov Chain Monte Carlo (MCMC) method are listed in **Table C6** (no vaccine protection) to **Table C11** (90% vaccine protection). **Table C12** summarizes the number of final cases of these scenarios along with the number of final cases when there are five initial infective cases.

**Figure C1. COVID-19 outbreak on the Panshi fast combat support ship.**

**(a)The epidemic curve of the COVID-19 outbreak on the ship**

**
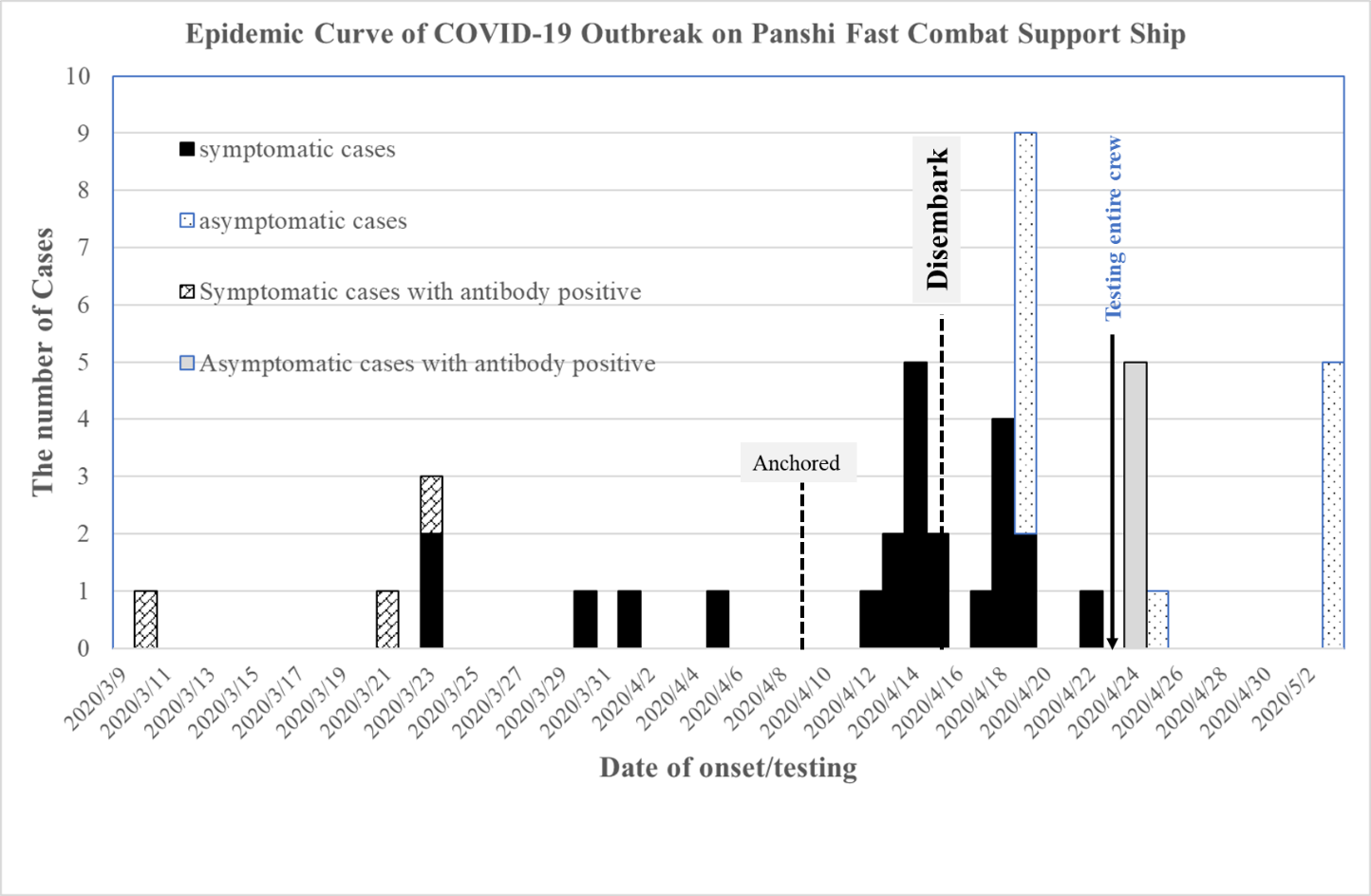
**

**Note.** Thirteen asymptomatic cases with RT-PCR positive, 4 asymptomatic cases with antibody positive, and one symptomatic case with antibody positive, without exact onset date, recorded by testing date.

**(b) The cumulated COVID-19 observed cases and simulated cumulated cases by SEIR model^*^**

**
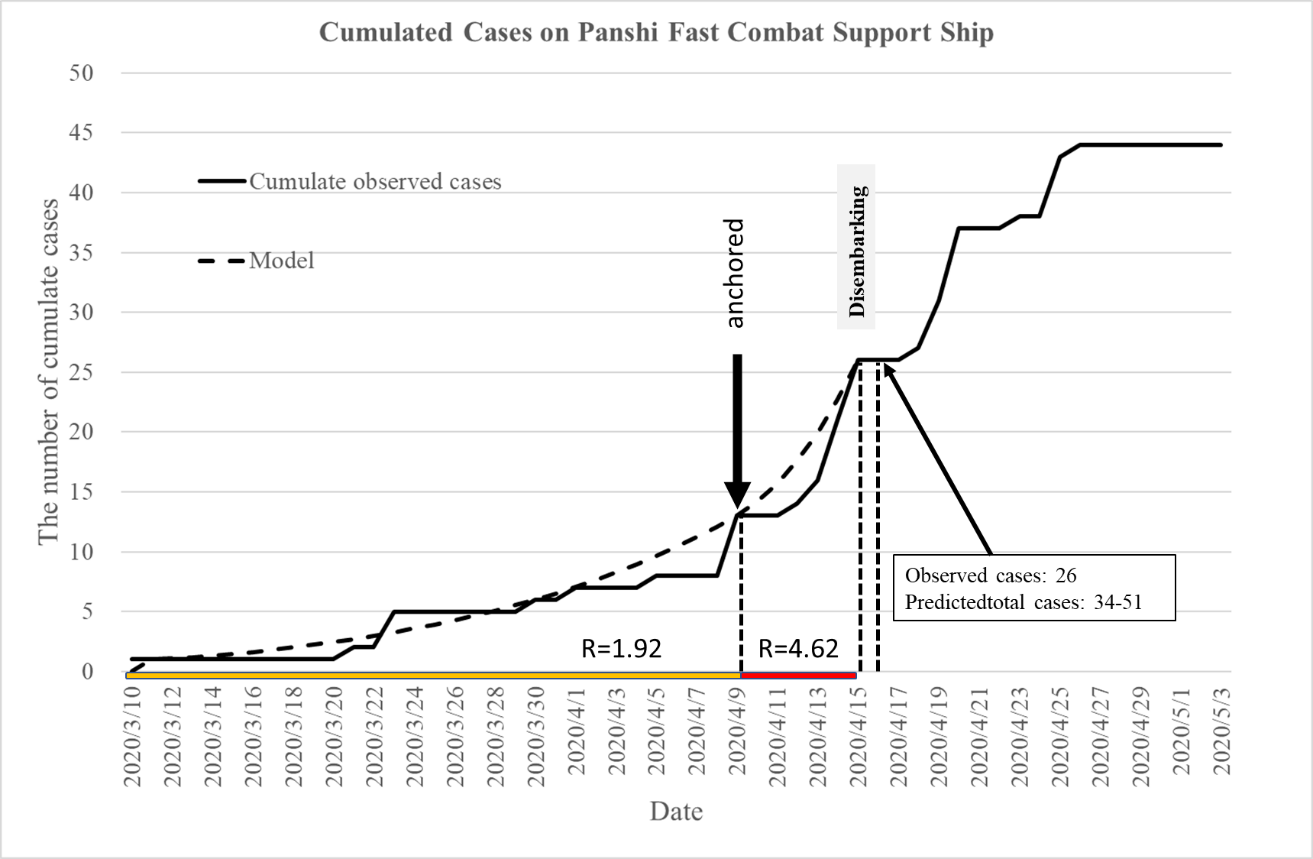
**

*R: Basic reproductive number

**Figure C2. The cumulated COVID-19 observed cases and simulated cumulated cases by deterministic SEIR model on warships.^*^**

**(a) COVID-19 outbreak on the USS Theodore Roosevelt aircraft carrier**

**
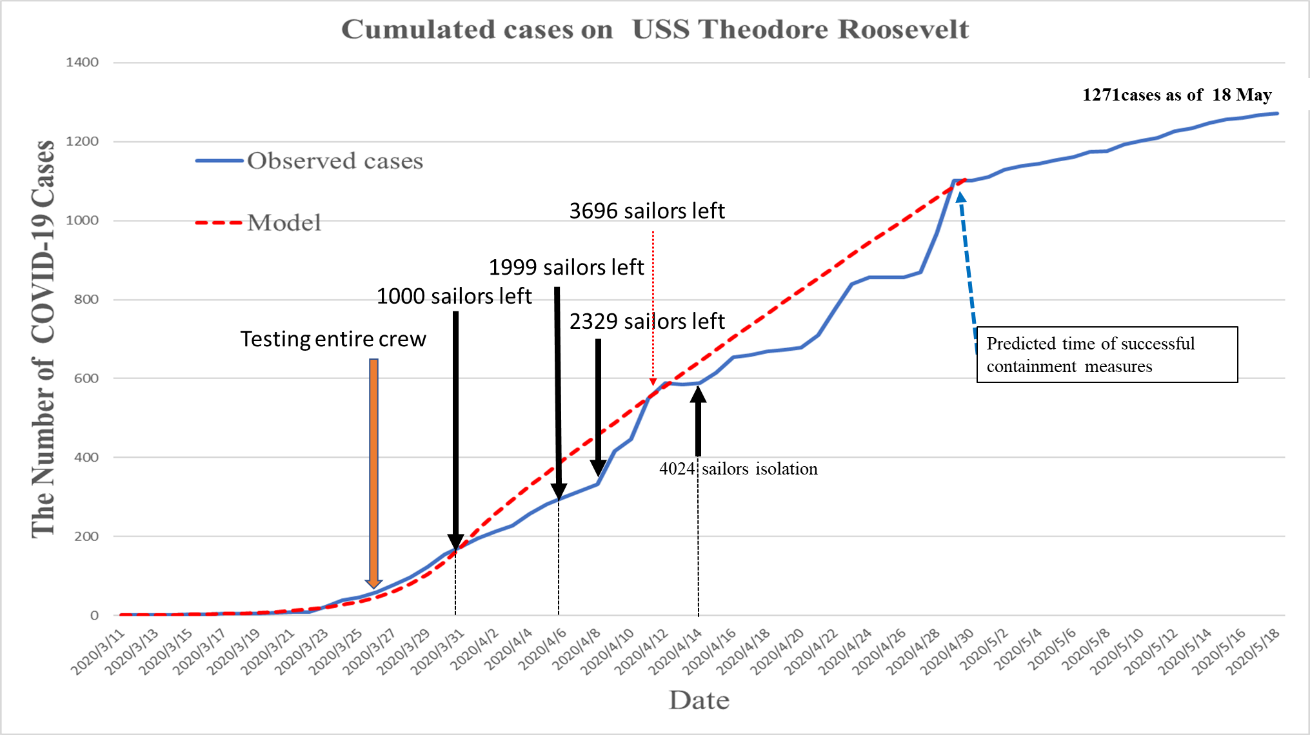
**

**(b) COVID-19 outbreak on the French Charles de Gaulle aircraft carrier**

**
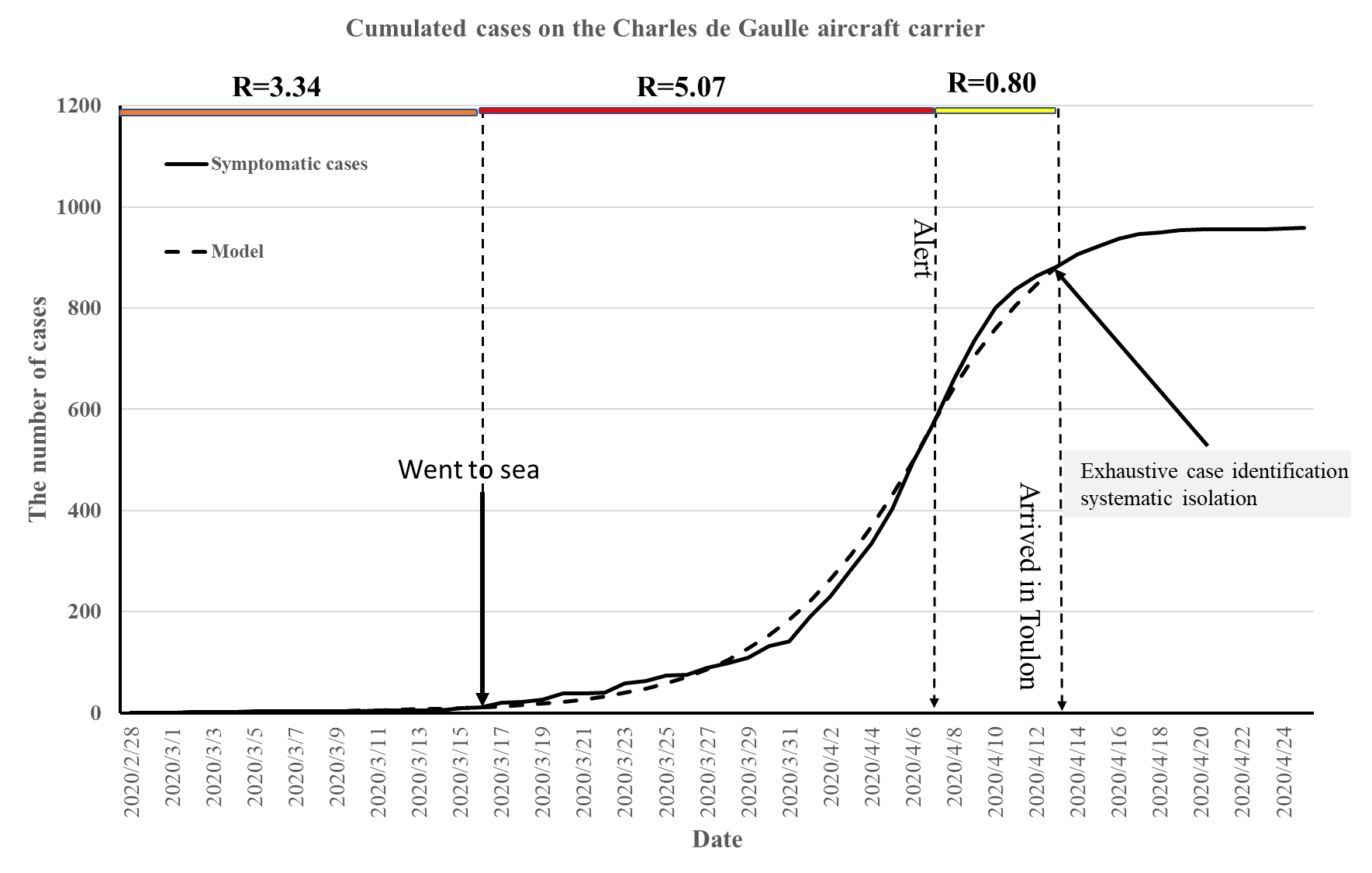
**

*****R: Basic reproductive number

**Figure C3. The cumulated COVID-19 observed cases and simulated cumulated cases by deterministic SEIR model on cruise ships.**

**(a) COVID-19 outbreak on the Diamond Princess Cruise Ship**

**
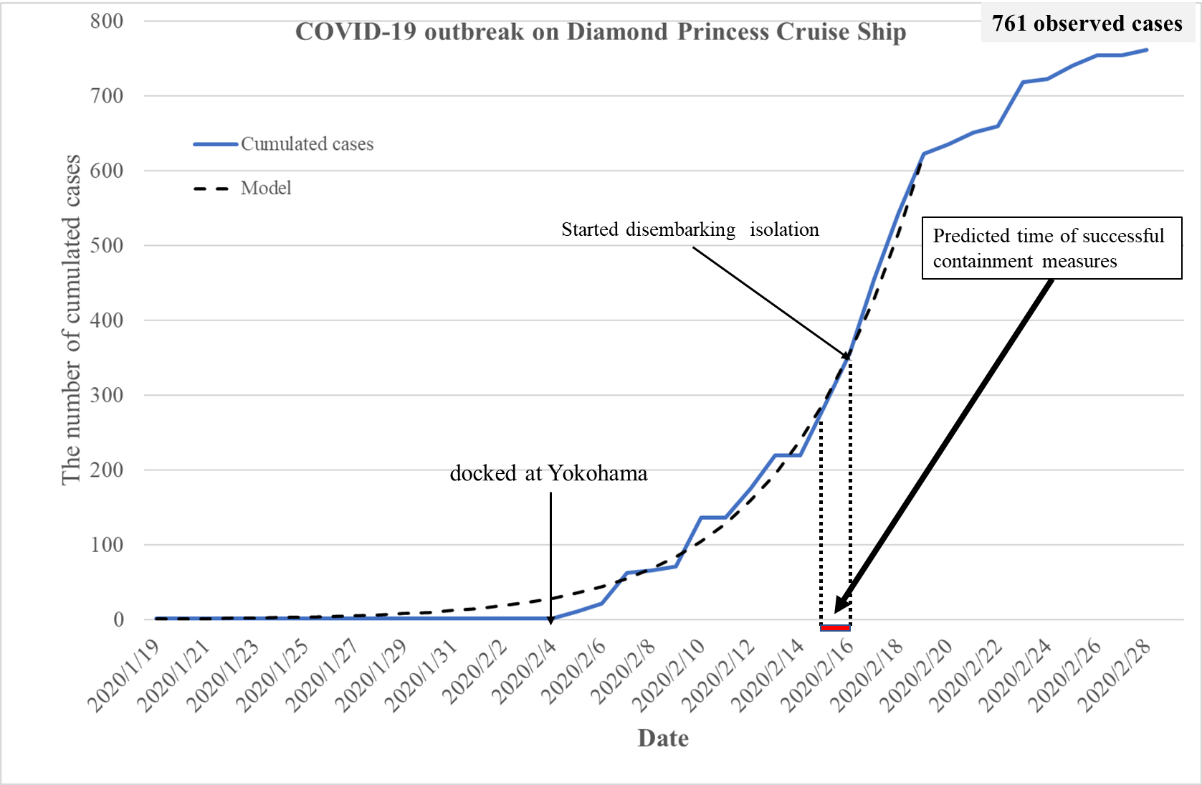
**

**(b) COVID-19 outbreak on the Grand Princess Ship**


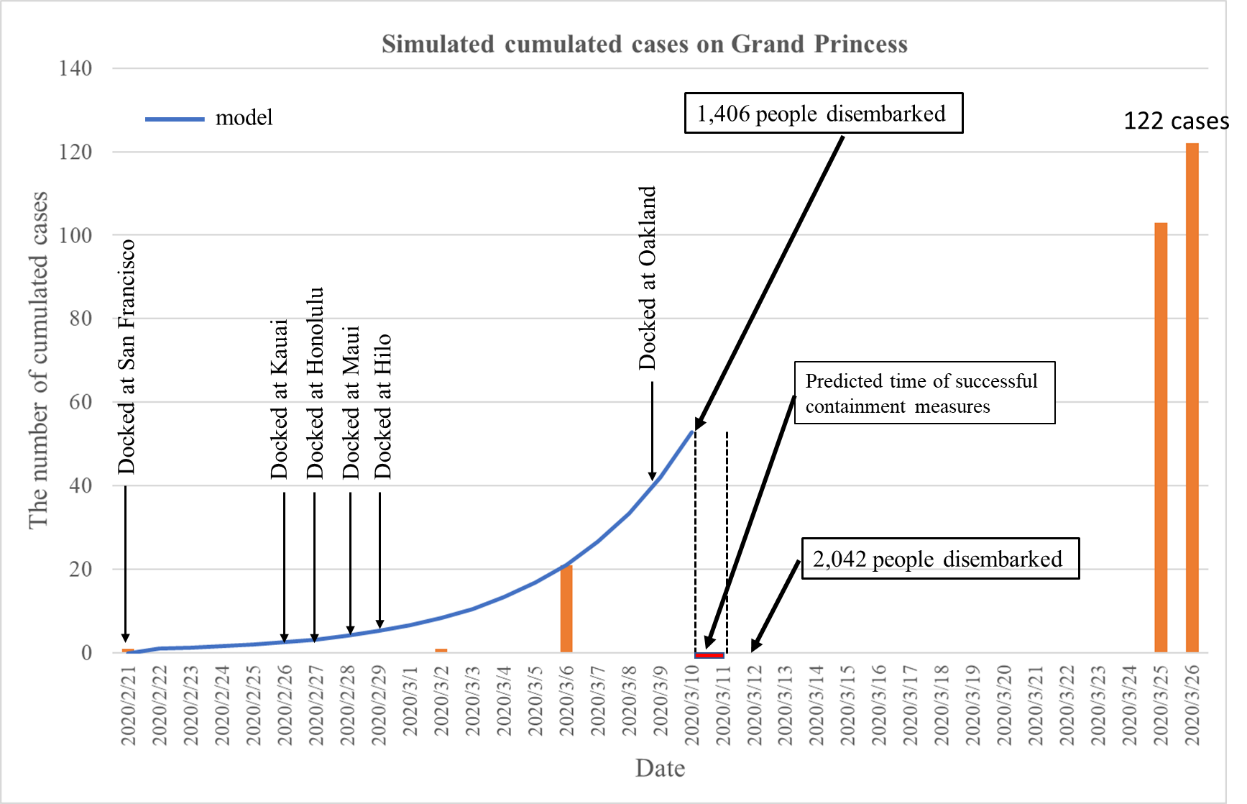


**(c) COVID-19 outbreak on the Greg Mortimer, polar cruise expedition ship**


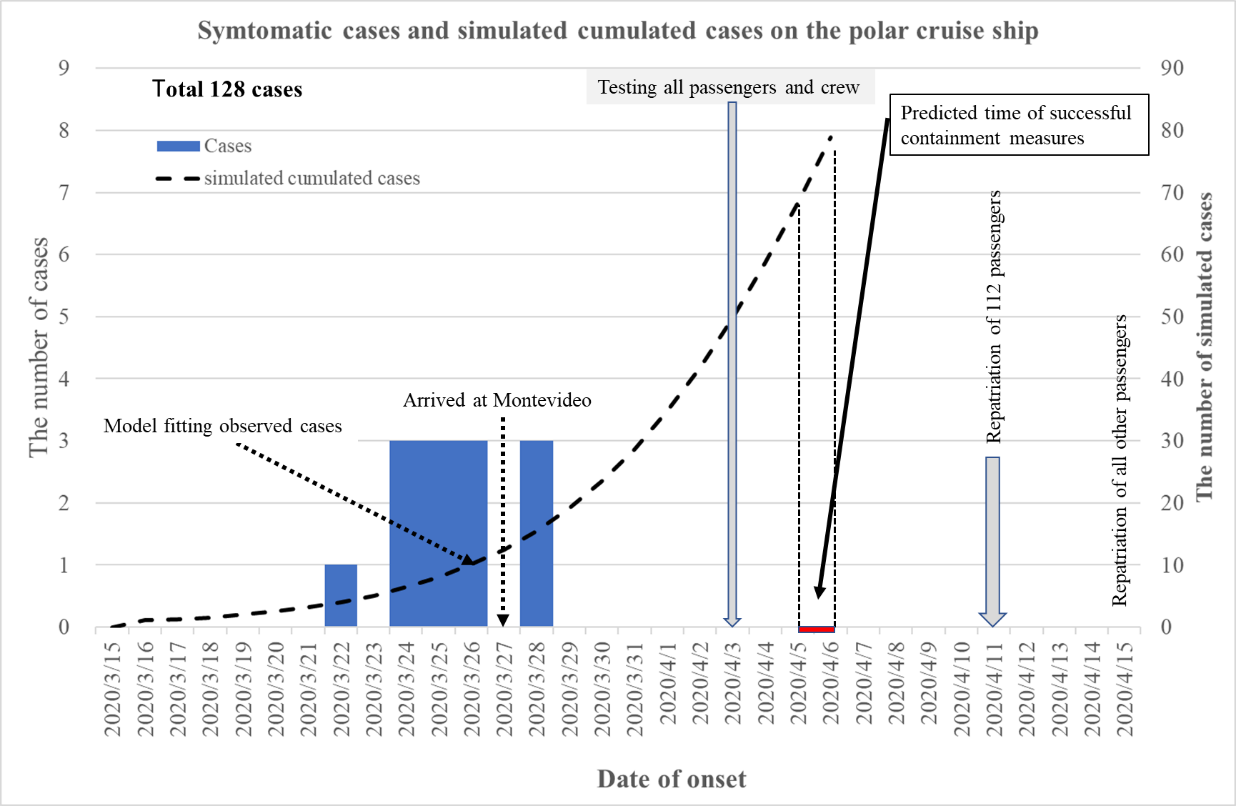


**Table C1. Estimated results on the parameters of COVID-19 transmission on each ship by using deterministic SEIR model.**

| **Name of ships** | **Estimated by deterministic SEIR model** | | |
| --- | --- | --- | --- |
|  | **β** | **R_0_** | **The time of successful containment measures (days)** |
| Panshi fast combat support ship | 0.291  (before 9 April)  0.660  (10-15 April) | 2.04  4.62 | 37  (15 April) |
| Theodore Roosevelt aircraft carrier | 0.972  (before 31 March)  0.173  (1-11 April) | 6.80  1.21 | 50-51  (29-30 April) |
| Charles de Gaulle aircraft carrier | 0.477  (before 16 March)  0.724  (17 March to 7 April)  0.115  (8-13 April) | 3.34  (before 16 March)  5.07  (17 March to 7 April)  0.80  (8-13 April) | 48  (15 April) |
| Diamond Princess Cruise Ship | 0.788 | 5.52 | 27-28  (15-16 February) |
| Greg Mortimer polar expedition cruise ship | 0.792 | 5.55 | 22-23  (5-6 April) |
| Grand Princess Cruise Ship | 0.796 | 5.58 | 19-20  (10-11 March) |

R_0_: basic reproductive number; SEIR model: Susceptible-Exposed-Infected-Recovered model.

**Table C2. The number of COVID-19 cumulated cases and simulated cumulated cases by using deterministic SEIR model on warships.**

|  | **USS Theodore Roosevelt**  **aircraft carrier (N=4779)** | | | | |  | **French Charles de Gaulle aircraft carrier (N=1767)** | | | | **Panshi fast combat**  **support ship (N=377)** | | | |
| --- | --- | --- | --- | --- | --- | --- | --- | --- | --- | --- | --- | --- | --- | --- |
| Day | Date | CC | | Model | | Day | Date | CC | Model | | Date | CC | Model | |
|  |  | S-cases | R-cases | I+R | E |  |  |  | I+R | E |  |  | I+R | E |
| 1 | 3/11 | 1 |  | 1 | 0 | 1 | 2/28 | 1 | 1.0 | 0.0 | 3/10 | 1 | 1 | 0 |
| 5 | 3/15 | 3 |  | 2.3 | 3.1 | 2 | 2/29 | 1 | 1.0 | 0.5 | 3/11 | 1 | 1 | 0 |
| 9 | 3/19 | 4 |  | 6.9 | 9.6 | 3 | 3/1 | 1 | 1.2 | 0.7 | 3/12 | 1 | 1 | 0 |
| 10 | 3/20 | 7 |  | 9.1 | 12.5 | 4 | 3/2 | 2 | 1.3 | 1.0 | 3/13 | 1 | 1 | 1 |
| 11 | 3/21 | 9 |  | 12 | 16.4 | 5 | 3/3 | 2 | 1.5 | 1.3 | 3/14 | 1 | 1 | 1 |
| 12 | 3/22 | 9 | 1 | 15.8 | 21.5 | 6 | 3/4 | 2 | 1.8 | 1.5 | 3/15 | 1 | 1 | 1 |
| 13 | 3/23 | 22 | 1 | 20.7 | 28.1 | 7 | 3/5 | 3 | 2.1 | 1.8 | 3/16 | 1 | 2 | 1 |
| 14 | 3/24 | 38 | 3 | 27.1 | 36.7 | 8 | 3/6 | 3 | 2.5 | 2.0 | 3/17 | 1 | 2 | 1 |
| 15 | 3/25 | 46 | 8 | 35.6 | 47.9 | 9 | 3/7 | 3 | 2.9 | 2.4 | 3/18 | 1 | 2 | 1 |
| 16 | 3/26 | 60 | 8 | 46.6 | 62.5 | 10 | 3/8 | 3 | 3.4 | 2.7 | 3/19 | 1 | 2 | 1 |
| 17 | 3/27 | 77 | 33 | 60.9 | 81.3 | 11 | 3/9 | 3 | 4.0 | 3.1 | 3/20 | 1 | 2 | 1 |
| 18 | 3/28 | 97 | 33 | 79.5 | 105.6 | 12 | 3/10 | 4 | 4.7 | 3.5 | 3/21 | 2 | 3 | 1 |
| 19 | 3/29 | 123 | 33 | 103.7 | 136.7 | 13 | 3/11 | 5 | 5.4 | 4.1 | 3/22 | 2 | 3 | 1 |
| 20 | 3/30 | 155 | 33 | 134.9 | 176.4 | 14 | 3/12 | 5 | 6.3 | 4.7 | 3/23 | 5 | 3 | 2 |
| 21 | 3/31 | 175 | 33 | **175** | 226.6 | 15 | 3/13 | 5 | 7.3 | 5.3 | 3/24 | 5 | 4 | 2 |
| 22 | 4/1 | 195 | 93 | 217.8 | 203.2 | 16 | 3/14 | 5 | 8.5 | 6.1 | 3/25 | 5 | 4 | 2 |
| 23 | 4/2 | 212 | 114 | 256.8 | 187.5 | 17 | 3/15 | 10 | 9.8 | 7.0 | 3/26 | 5 | 4 | 2 |
| 24 | 4/3 | 227 | 137 | 293.2 | 176.7 | 18 | 3/16 | 11 | 11.3 | 8.0 | 3/27 | 5 | 5 | 2 |
| 25 | 4/4 | 258 | 155 | 327.8 | 169.3 | 19 | 3/17 | 20 | 13.1 | 10.7 | 3/28 | 5 | 5 | 2 |
| 26 | 4/5 | 282 | 155 | 361.1 | 164.3 | 20 | 3/18 | 22 | 15.6 | 13.5 | 3/29 | 5 | 6 | 2 |
| 27 | 4/6 | 299 | 172 | 393.7 | 160.8 | 21 | 3/19 | 27 | 18.6 | 16.8 | 3/30 | 6 | 6 | 2 |
| 28 | 4/7 | 316 | 230 | 425.6 | 158.5 | 22 | 3/20 | 38 | 22.4 | 20.8 | 3/31 | 6 | 7 | 3 |
| 29 | 4/8 | 333 | 286 | 457.1 | 157.0 | 23 | 3/21 | 38 | 27.0 | 25.6 | 4/1 | 7 | 7 | 3 |
| 30 | 4/9 | 340 | 416 | 488.4 | 155.9 | 24 | 3/22 | 40 | 32.7 | 31.4 | 4/2 | 7 | 8 | 3 |
| 31 | 4/10 | 349 | 447 | 519.6 | 155.2 | 25 | 3/23 | 59 | 39.7 | 38.4 | 4/3 | 7 | 8 | 3 |
| 32 | 4/11 | 359 | 550 | **550.5** | 154.6 | 26 | 3/24 | 63 | 48.2 | 46.9 | 4/4 | 7 | 9 | 3 |
| 33 | 4/12 | 369 | 585 | 581.4 | 154.2 | 27 | 3/25 | 74 | 58.6 | 57.1 | 4/5 | 8 | 10 | 4 |
| 34 | 4/13 | 415 | 585 | 612.2 | 153.8 | 28 | 3/26 | 75 | 71.2 | 69.3 | 4/6 | 8 | 10 | 4 |
| 35 | 4/14 | 439 | 589 | 642.9 | 153.4 | 29 | 3/27 | 89 | 86.5 | 83.7 | 4/7 | 8 | 11 | 4 |
| 36 | 4/15 | 471 | 615 | 673.6 | 152.9 | 30 | 3/28 | 98 | 104.9 | 100.8 | 4/8 | 8 | 12 | 5 |
| 37 | 4/16 | 481 | 655 | 704.1 | 152.4 | 31 | 3/29 | 109 | 127.0 | 120.6 | 4/9 | 13* | 13 | 5 |
| 38 | 4/17 | 487 | 660 | 734.5 | 151.9 | 32 | 3/30 | 132 | 153.4 | 143.3 | 4/10 | 13 | 14 | 7 |
| 39 | 4/18 | 499 | 669 | 764.8 | 151.3 | 33 | 3/31 | 141 | 184.6 | 169.0 | 4/11 | 13 | 16 | 9 |
| 40 | 4/19 | 508 | 672 | 795 | 150.6 | 34 | 4/1 | 190 | 221.2 | 197.6 | 4/12 | 14 | 18 | 10 |
| 41 | 4/20 | 513 | 678 | 825 | 149.8 | 35 | 4/2 | 230 | 263.8 | 228.5 | 4/13 | 16 | 20 | 13 |
| 42 | 4/21 | 522 | 710 | 854.9 | 148.9 | 36 | 4/3 | 284 | 312.8 | 261.0 | 4/14 | 21 | 23 | 15 |
| 43 | 4/22 | 525 | 777 | 884.6 | 148.0 | 37 | 4/4 | 334 | 368.3 | 294.3 | 4/15 | 26* | 26 | 18 |
| 44 | 4/23 | 532 | 840 | 914 | 147.0 | 38 | 4/5 | 404 | 430.4 | 326.9 |  |  |  |  |
| 45 | 4/24 | 542 | 856 | 943.3 | 145.9 | 39 | 4/6 | 497 | 498.8 | 357.0 |  |  |  |  |
| 46 | 4/25 | 545 | 856 | 972.3 | 144.7 | 40 | 4/7 | 573 | 572.9 | 383.3 |  |  |  |  |
| 47 | 4/26 | 550 | 856 | 1001.1 | 143.4 | 41 | 4/8 | 661 | 643.7 | 327.9 |  |  |  |  |
| 48 | 4/27 | 556 | 969 | 1029.6 | 142.1 | 42 | 4/9 | 737 | 704.8 | 283.8 |  |  |  |  |
| 49 | 4/28 | 557 | 1102 | 1057.9 | 140.7 | 43 | 4/10 | 800 | 757.9 | 247.9 |  |  |  |  |
| 50 | 4/29 | 564 |  | 1085.9 | 139.2 | 44 | 4/11 | 837 | 804.5 | 218.0 |  |  |  |  |
| 51 | 4/30 | 567 |  | 1113.6 | 137.7 | 45 | 4/12 | 863 | 845.6 | 193.1 |  |  |  |  |
|  |  |  |  |  |  | 46 | 4/13 | 882 | 882.1 | 172.1 |  |  |  |  |
|  |  |  |  |  |  | 47 | 4/14 | 906 | 914.7 | 207.1 |  |  |  |  |
|  |  |  |  |  |  | 48 | 4/15 | 922 | 944.0 | 205.2 |  |  |  |  |
|  |  |  |  |  |  | 49 | 4/16 | 937 | 970.3 | 204.5 |  |  |  |  |

E: exposed state; I:infectious state; R: recovery state; CC: cumulated cases; S-cases: cases by symptomatic onset date; R-cases: cases by reported date * We suppose that 5 asymptomatic cases with antibody positive occurred before April 9 and half of asymptomatic cases testing positive on April 19 which occurred on April 15.

**Table C3. The number of COVID-19 cumulated cases and simulated cumulated cases by deterministic SEIR model on cruise ships.**

|  | **Diamond Princess**  **(N=3711)** | | | | **Grand Princess**  **(N=3571)** | | | | **Greg Mortimer polar expedition cruise ship**  **(N=217)** | | | |
| --- | --- | --- | --- | --- | --- | --- | --- | --- | --- | --- | --- | --- |
| Day | Date | Cumulated Cases | Model | | Date | Cumulated Cases | Model | | Date | Cumulated Cases | Model | |
|  |  |  | I+R | E |  |  | I+R | E |  |  | I+R | E |
| 1 | 1/20 | 1 | 1.0 | 0.0 | 2/21 | 1 | 1 | 0.0 | 3/15 |  | 1.0 | 0.0 |
| 2 | 1/21 | 1 | 1.1 | 0.7 | 2/22 |  | 1.1 | 0.7 | 3/16 |  | 1.1 | 0.7 |
| 3 | 1/22 | 1 | 1.3 | 1.2 | 2/23 |  | 1.3 | 1.3 | 3/17 |  | 1.3 | 1.2 |
| 4 | 1/23 | 1 | 1.6 | 1.8 | 2/24 |  | 1.6 | 1.8 | 3/18 |  | 1.6 | 1.8 |
| 5 | 1/24 | 1 | 2.0 | 2.3 | 2/25 |  | 2.0 | 2.4 | 3/19 |  | 2.0 | 2.3 |
| 6 | 1/25 | 1 | 2.5 | 3.0 | 2/26 |  | 2.5 | 3.1 | 3/20 |  | 2.5 | 3.0 |
| 7 | 1/26 | 1 | 3.2 | 3.8 | 2/27 |  | 3.2 | 3.9 | 3/21 |  | 3.2 | 3.8 |
| 8 | 1/27 | 1 | 4.1 | 4.8 | 2/28 |  | 4.1 | 4.9 | 3/22 | 1 | 4.0 | 4.7 |
| 9 | 1/28 | 1 | 5.1 | 6.0 | 2/29 |  | 5.2 | 6.2 | 3/23 | 1 | 5.1 | 5.9 |
| 10 | 1/29 | 1 | 6.5 | 7.6 | 3/1 |  | 6.6 | 7.8 | 3/24 | 4 | 6.4 | 7.3 |
| 11 | 1/30 | 1 | 8.2 | 9.5 | 3/2 | 1 | 8.4 | 9.8 | 3/25 | 7 | 8.0 | 9.0 |
| 12 | 1/31 | 1 | 10.3 | 11.9 | 3/3 |  | 10.6 | 12.3 | 3/26 | 10 | 10.1 | 11.1 |
| 13 | 2/1 | 1 | 13.0 | 14.9 | 3/4 |  | 13.3 | 15.4 | 3/27 | 10 | 12.5 | 13.6 |
| 14 | 2/2 | 1 | 16.4 | 18.7 | 3/5 |  | 16.8 | 19.4 | 3/28 | 13 | 15.5 | 16.5 |
| 15 | 2/3 | 1 | 20.6 | 23.4 | 3/6 | 21 | 21.2 | 24.3 | 3/29 | 13 | 19.2 | 19.9 |
| 16 | 2/4 | 1 | 25.8 | 29.2 | 3/7 |  | 26.6 | 30.4 | 3/30 | 13 | 23.5 | 23.8 |
| 17 | 2/5 | 11 | 32.4 | 36.5 | 3/8 |  | 33.5 | 38.1 | 3/31 | 13 | 28.7 | 28.0 |
| 18 | 2/6 | 21 | 40.6 | 45.6 | 3/9 |  | 42.1 | 47.6 | 4/1 | 13 | 34.8 | 32.7 |
| 19 | 2/7 | 62 | 50.8 | 56.9 | 3/10 |  | 52.8 | 59.5 | 4/2 | 13 | 41.8 | 37.5 |
| 20 | 2/8 | 65 | 63.6 | 70.8 | 3/11 |  | 66.0 | 74.1 | 4/3 | 21 | 49.8 | 42.3 |
| 21 | 2/9 | 71 | 79.4 | 87.9 | 3/12 |  | 82.6 | 92.2 | 4/4 | 22 | 58.7 | 46.7 |
| 22 | 2/10 | 136 | 99.1 | 108.9 | 3/13 |  |  |  | 4/5 | 25 | 68.4 | 50.6 |
| 23 | 2/11 | 136 | 123.4 | 134.5 | 3/14 |  |  |  | 4/6 | 26 | 78.9 | 53.7 |
| 24 | 2/12 | 175 | 153.3 | 165.5 | 3/15 |  |  |  | 4/7 | 27 | 89.8 | 55.6 |
| 25 | 2/13 | 219 | 190.1 | 202.7 | 3/16 |  |  |  |  | (128*) |  |  |
| 26 | 2/14 | 219 | 235.0 | 246.8 | 3/17 |  |  |  |  |  |  |  |
| 27 | 2/15 | 286 | 289.5 | 298.5 | 3/18 |  |  |  |  |  |  |  |
| 28 | 2/16 | 356 | 355.0 | 358.2 | 3/19 |  |  |  |  |  |  |  |
| 29 | 2/17 | 455 | 431.8 | 411.3 | 3/20 |  |  |  |  |  |  |  |
| 30 | 2/18 | 557 | 520.1 | 472.1 | 3/21 |  |  |  |  |  |  |  |
| 31 | 2/19 | 622 | 621.0 | 537.6 | 3/22 |  |  |  |  |  |  |  |
| 32 | 2/20 | 635 |  |  | 3/23 |  |  |  |  |  |  |  |
| 33 | 2/21 | 651 |  |  | 3/24 |  |  |  |  |  |  |  |
| 34 | 2/22 | 659 |  |  | 3/25 | 103 |  |  |  |  |  |  |
| 35 | 2/23 | 718 |  |  | 3/26 | 122 |  |  |  |  |  |  |
| 36 | 2/24 | 722 |  |  |  |  |  |  |  |  |  |  |
| 37 | 2/25 | 740 |  |  |  |  |  |  |  |  |  |  |
| 38 | 2/26 | 706 |  |  |  |  |  |  |  |  |  |  |
| 39 | 2/27 | 706 |  |  |  |  |  |  |  |  |  |  |
| 40 | 2/28 | 761 |  |  |  |  |  |  |  |  |  |  |

E: exposed state; I: infectious state; R: recovery state. Cumulated cases included one index case. Cases were confirmed in Japan and outside Japan on Diamond Princess cruise ship.

**Table C4. Total number of COVID-19 potential cases predicted by Bayesian SEIR model on the warship of USS Theodore Roosevelt, French Charles de Gaulle aircraft carrier, and Panshi fast combat support ship.**

|  | **USS Theodore Roosevelt**  **aircraft carrier (N=4779)** | | | | **French Charles de Gaulle aircraft carrier (N=1767)** | | | | **Panshi fast combat**  **support ship (N=377)** | | | |
| --- | --- | --- | --- | --- | --- | --- | --- | --- | --- | --- | --- | --- |
| Day | Date | E+I+R  prediction | 95% CI | | Date | E+I+R  prediction | 95% CI | | Date | E+I+R  prediction | 95% CI | |
| 1 | 3/11 | 1.9 | 1.9 | 1.9 | 2/28 | 1.4 | 1.4 | 1.5 | 3/10 | 1.3 | 1.2 | 1.3 |
| 2 | 3/12 | 2.8 | 2.7 | 2.8 | 2/29 | 1.9 | 1.8 | 1.9 | 3/11 | 1.5 | 1.5 | 1.5 |
| 3 | 3/13 | 3.8 | 3.7 | 3.8 | 3/1 | 2.3 | 2.2 | 2.3 | 3/12 | 1.7 | 1.7 | 1.8 |
| 4 | 3/14 | 5.0 | 4.9 | 5.1 | 3/2 | 2.7 | 2.6 | 2.8 | 3/13 | 1.9 | 1.9 | 2.0 |
| 5 | 3/15 | 6.6 | 6.5 | 6.7 | 3/3 | 3.2 | 3.1 | 3.3 | 3/14 | 2.2 | 2.1 | 2.2 |
| 6 | 3/16 | 8.5 | 8.4 | 8.7 | 3/4 | 3.8 | 3.6 | 3.9 | 3/15 | 2.4 | 2.3 | 2.5 |
| 7 | 3/17 | 11.0 | 10.8 | 11.2 | 3/5 | 4.4 | 4.2 | 4.6 | 3/16 | 2.6 | 2.5 | 2.8 |
| 8 | 3/18 | 14.2 | 13.9 | 14.5 | 3/6 | 5.1 | 4.8 | 5.3 | 3/17 | 2.9 | 2.7 | 3.0 |
| 9 | 3/19 | 18.4 | 17.9 | 18.8 | 3/7 | 5.8 | 5.5 | 6.2 | 3/18 | 3.1 | 2.9 | 3.3 |
| 10 | 3/20 | 23.7 | 23.1 | 24.2 | 3/8 | 6.7 | 6.3 | 7.2 | 3/19 | 3.4 | 3.2 | 3.6 |
| 11 | 3/21 | 30.4 | 29.7 | 31.2 | 3/9 | 7.8 | 7.2 | 8.3 | 3/20 | 3.7 | 3.4 | 4.0 |
| 12 | 3/22 | 39.2 | 38.1 | 40.2 | 3/10 | 8.9 | 8.3 | 9.5 | 3/21 | 4.0 | 3.7 | 4.3 |
| 13 | 3/23 | 50.3 | 48.9 | 51.8 | 3/11 | 10.3 | 9.4 | 11.0 | 3/22 | 4.3 | 3.9 | 4.7 |
| 14 | 3/24 | 64.7 | 62.7 | 66.7 | 3/12 | 11.8 | 10.8 | 12.7 | 3/23 | 4.7 | 4.2 | 5.1 |
| 15 | 3/25 | 83.0 | 80.2 | 85.6 | 3/13 | 13.5 | 12.3 | 14.6 | 3/24 | 5.0 | 4.5 | 5.5 |
| 16 | 3/26 | 106.4 | 102.6 | 109.9 | 3/14 | 15.5 | 14.0 | 16.9 | 3/25 | 5.4 | 4.9 | 6.0 |
| 17 | 3/27 | 136.2 | 131.5 | 141.3 | 3/15 | 17.8 | 15.9 | 19.4 | 3/26 | 5.9 | 5.2 | 6.5 |
| 18 | 3/28 | 174.1 | 167.2 | 180.3 | 3/16 | 20.3 | 18.1 | 22.3 | 3/27 | 6.3 | 5.6 | 7.0 |
| 19 | 3/29 | 222.1 | 213.0 | 230.3 | 3/17 | 24.9 | 22.4 | 27.2 | 3/28 | 6.8 | 5.9 | 7.5 |
| 20 | 3/30 | 282.7 | 271.4 | 294.3 | 3/18 | 30.4 | 27.4 | 33.0 | 3/29 | 7.3 | 6.4 | 8.2 |
| 21 | 3/31 | 358.7 | 343.8 | 373.7 | 3/19 | 36.9 | 33.6 | 40.0 | 3/30 | 7.8 | 6.8 | 8.8 |
| 22 | 4/1 | 376.3 | 362.0 | 390.8 | 3/20 | 44.8 | 40.9 | 48.2 | 3/31 | 8.4 | 7.2 | 9.5 |
| 23 | 4/2 | 396.7 | 383.5 | 411.1 | 3/21 | 54.4 | 50.1 | 58.3 | 4/1 | 9.0 | 7.7 | 10.2 |
| 24 | 4/3 | 418.9 | 405.5 | 431.8 | 3/22 | 66.1 | 61.2 | 70.5 | 4/2 | 9.6 | 8.2 | 11.0 |
| 25 | 4/4 | 442.6 | 430.3 | 455.3 | 3/23 | 80.3 | 75.1 | 85.6 | 4/3 | 10.3 | 8.7 | 11.8 |
| 26 | 4/5 | 467.3 | 455.2 | 479.0 | 3/24 | 97.5 | 91.5 | 103.2 | 4/4 | 11.0 | 9.2 | 12.7 |
| 27 | 4/6 | 492.8 | 481.7 | 504.7 | 3/25 | 118.2 | 111.7 | 124.6 | 4/5 | 11.8 | 9.8 | 13.6 |
| 28 | 4/7 | 518.7 | 508.0 | 530.2 | 3/26 | 143.1 | 135.8 | 149.8 | 4/6 | 12.6 | 10.4 | 14.7 |
| 29 | 4/8 | 545.0 | 533.8 | 556.1 | 3/27 | 173.0 | 165.4 | 180.3 | 4/7 | 13.4 | 11.0 | 15.7 |
| 30 | 4/9 | 571.6 | 559.7 | 582.6 | 3/28 | 208.4 | 200.3 | 216.2 | 4/8 | 14.3 | 11.7 | 16.9 |
| 31 | 4/10 | 598.4 | 585.9 | 610.1 | 3/29 | 250.3 | 241.4 | 258.3 | 4/9 | 15.3 | 12.4 | 18.1 |
| 32 | 4/11 | 625.3 | 611.7 | 638.2 | 3/30 | 299.2 | 289.6 | 308.9 | 4/10 | 17.7 | 15.1 | 20.6 |
| 33 | 4/12 | 652.2 | 637.6 | 666.8 | 3/31 | 355.9 | 344.1 | 366.3 | 4/11 | 20.4 | 17.6 | 23.1 |
| 34 | 4/13 | 679.2 | 663.0 | 695.3 | 4/1 | 420.6 | 407.4 | 433.3 | 4/12 | 23.5 | 20.7 | 26.5 |
| 35 | 4/14 | 706.2 | 687.7 | 723.9 | 4/2 | 493.6 | 477.4 | 508.8 | 4/13 | 27.1 | 23.8 | 30.7 |
| 36 | 4/15 | 733.1 | 712.4 | 753.4 | 4/3 | 574.4 | 557.3 | 595.3 | 4/14 | 31.3 | 26.9 | 36.0 |
| 37 | 4/16 | 760.0 | 738.2 | 784.4 | 4/4 | 662.4 | 638.8 | 684.3 | 4/15 | 36.3 | 30.6 | 43.2 |
| 38 | 4/17 | 786.8 | 761.7 | 813.3 | 4/5 | 756.1 | 728.3 | 781.7 | **4/16** | 42.1 | 33.7 | 51.4 |
| 39 | 4/18 | 813.5 | 786.7 | 844.6 | 4/6 | 853.7 | 819.8 | 882.1 | - | - | - | - |
| 40 | 4/19 | 840.1 | 807.6 | 872.0 | 4/7 | 952.9 | 919.7 | 989.8 | - | - | - | - |
| 41 | 4/20 | 866.6 | 830.3 | 901.1 | 4/8 | 986.2 | 966.0 | 1010.5 | - | - | - | - |

E: exposed state; I: infectious state; R: recovery state. CI: credible interval

**Table C4. Total number of COVID-19 potential cases predicted by Bayesian SEIR model on the warship of USS Theodore Roosevelt, French Charles de Gaulle aircraft carrier, and Panshi fast combat support ship.** (continue)

|  | **USS Theodore Roosevelt**  **aircraft carrier (N=4779)** | | | | **French Charles de Gaulle aircraft carrier (N=1767)** | | | |
| --- | --- | --- | --- | --- | --- | --- | --- | --- |
| Day | Date | E+I+R  prediction | 95% CI | | Date | E+I+R  prediction | 95% CI | |
| 42 | 4/21 | 892.9 | 853.3 | 930.8 | 4/9 | 1020.3 | 993.5 | 1048.6 |
| 43 | 4/22 | 919.1 | 876.5 | 961.2 | 4/10 | 1054.2 | 1007.0 | 1099.7 |
| 44 | 4/23 | 945.1 | 900.0 | 992.1 | 4/11 | 1087.1 | 1020.7 | 1153.4 |
| 45 | 4/24 | 970.9 | 923.0 | 1022.1 | 4/12 | 1118.7 | 1034.5 | 1204.7 |
| 46 | 4/25 | 996.6 | 945.9 | 1052.9 | 4/13 | 1148.6 | 1047.3 | 1252.7 |
| 47 | 4/26 | 1022.0 | 966.5 | 1081.3 | 4/14 | 1176.7 | 1057.4 | 1297.2 |
| 48 | 4/27 | 1047.2 | 988.1 | 1111.0 | 4/15 | 1202.9 | 1058.7 | 1330.1 |
| 49 | 4/28 | 1072.1 | 1009.5 | 1140.8 | 4/16 | 1227.2 | 1067.7 | 1366.8 |
| 50 | 4/29 | 1096.8 | 1030.2 | 1170.1 | 4/17 | 1249.8 | 1089.4 | 1413.0 |
| 51 | 4/30 | 1121.3 | 1049.2 | 1197.5 | 4/18 | 1270.5 | 1097.4 | 1442.9 |
| 52 | 5/1 | 1145.5 | 1069.0 | 1226.0 | 4/19 | 1289.6 | 1106.4 | 1471.8 |
| 53 | 5/2 | 1169.4 | 1087.2 | 1252.7 | 4/20 | 1307.2 | 1114.6 | 1497.4 |
| 54 | **5/3** | 1193.0 | 1109.1 | 1283.4 | 4/21 | 1323.2 | 1121.6 | 1519.5 |
| 55 | 5/4 | 1216.4 | 1125.1 | 1307.9 | 4/22 | 1337.9 | 1137.9 | 1548.5 |
| 56 | 5/5 | 1239.5 | 1145.2 | 1336.9 | 4/23 | 1351.4 | 1144.1 | 1565.7 |
| 57 | 5/6 | 1262.2 | 1163.3 | 1363.8 | 4/24 | 1363.7 | 1152.1 | 1582.9 |
| 58 | 5/7 | 1284.6 | 1180.7 | 1389.8 | - | - | - | - |
| 59 | 5/8 | 1306.8 | 1198.3 | 1416.0 | - | - | - | - |
| 60 | 5/9 | 1328.6 | 1217.4 | 1443.7 | - | - | - | - |

E: exposed state; I: infectious state; R: recovery state. CI: credible interval

**Table C5. Total number of COVID-19 potential cases predicted by Bayesian SEIR model on the cruise ship of Diamond Princess, Grand Princess, and Greg Mortimer.**

|  | **Diamond Princess**  **(N=3711)** | | | | **Grand Princess**  **(N=3571)** | | | | **Greg Mortimer**  **(N=217)** | | | |
| --- | --- | --- | --- | --- | --- | --- | --- | --- | --- | --- | --- | --- |
| Day | Date | E+I+R  prediction | 95% CI | | Date | E+I+R  prediction | 95% CI | | Date | E+I+R  prediction | 95% CI | |
| 1 | 1/20 | 1.8 | 1.6 | 1.9 | 2/21 | 1.7 | 1.7 | 1.7 | 3/15 | 1.7 | 1.5 | 1.8 |
| 2 | 1/21 | 2.5 | 2.2 | 2.8 | 2/22 | 2.4 | 2.3 | 2.5 | 3/16 | 2.3 | 2.0 | 2.6 |
| 3 | 1/22 | 3.3 | 2.9 | 3.8 | 2/23 | 3.1 | 3.0 | 3.2 | 3/17 | 3.0 | 2.5 | 3.5 |
| 4 | 1/23 | 4.3 | 3.7 | 4.8 | 2/24 | 4.0 | 3.8 | 4.2 | 3/18 | 3.8 | 3.0 | 4.5 |
| 5 | 1/24 | 5.5 | 4.7 | 6.2 | 2/25 | 5.0 | 4.8 | 5.2 | 3/19 | 4.7 | 3.6 | 5.8 |
| 6 | 1/25 | 6.9 | 6.0 | 7.8 | 2/26 | 6.2 | 5.9 | 6.6 | 3/20 | 5.8 | 4.2 | 7.2 |
| 7 | 1/26 | 8.6 | 7.5 | 9.8 | 2/27 | 7.7 | 7.2 | 8.1 | 3/21 | 7.1 | 4.9 | 9.1 |
| 8 | 1/27 | 10.8 | 9.3 | 12.3 | 2/28 | 9.5 | 8.8 | 10.1 | 3/22 | 8.7 | 5.7 | 11.5 |
| 9 | 1/28 | 13.5 | 11.7 | 15.5 | 2/29 | 11.6 | 10.7 | 12.4 | 3/23 | 10.6 | 6.7 | 14.4 |
| 10 | 1/29 | 16.9 | 14.5 | 19.3 | 3/1 | 14.2 | 13.0 | 15.3 | 3/24 | 12.9 | 7.8 | 17.9 |
| 11 | 1/30 | 21.1 | 18.0 | 24.2 | 3/2 | 17.3 | 15.8 | 18.8 | 3/25 | 15.6 | 9.1 | 22.2 |
| 12 | 1/31 | 26.3 | 22.5 | 30.4 | 3/3 | 21.1 | 19.1 | 23.0 | 3/26 | 18.9 | 10.6 | 27.6 |
| 13 | 2/1 | 32.8 | 28.0 | 38.0 | 3/4 | 25.5 | 23.0 | 28.0 | 3/27 | 22.8 | 12.2 | 33.8 |
| 14 | 2/2 | 40.8 | 34.6 | 47.4 | 3/5 | 30.8 | 27.7 | 34.0 | 3/28 | 27.4 | 14.2 | 41.4 |
| 15 | 2/3 | 50.8 | 42.8 | 59.2 | 3/6 | 37.0 | 33.1 | 40.9 | 3/29 | 32.7 | 16.4 | 50.1 |
| 16 | 2/4 | 63.1 | 52.9 | 73.7 | 3/7 | 44.1 | 39.3 | 48.9 | 3/30 | 38.8 | 18.8 | 59.9 |
| 17 | 2/5 | 78.4 | 65.4 | 92.0 | 3/8 | 52.2 | 46.6 | 58.1 | 3/31 | 45.8 | 21.6 | 71.1 |
| 18 | 2/6 | 97.4 | 81.5 | 115.4 | 3/9 | 61.4 | 54.7 | 68.3 | 4/1 | 53.7 | 25.0 | 83.7 |
| 19 | 2/7 | 120.7 | 99.1 | 142.3 | 3/10 | 71.5 | 63.5 | 79.3 | 4/2 | 62.4 | 29.5 | 97.9 |
| 20 | 2/8 | 149.4 | 124.1 | 178.6 | 3/11 | 82.5 | 74.0 | 92.0 | 4/3 | 71.9 | 33.7 | 111.8 |
| 21 | 2/9 | 184.7 | 152.0 | 220.8 | 3/12 | 94.2 | 84.7 | 104.6 | 4/4 | 82.0 | 39.2 | 126.4 |
| 22 | 2/10 | 227.8 | 187.3 | 273.7 | 3/13 | 106.4 | 96.0 | 117.5 | **4/5** | 92.6 | 44.5 | 140.0 |
| 23 | 2/11 | 280.2 | 229.5 | 337.3 | **3/14** | 118.7 | 107.7 | 130.3 | 4/6 | 103.5 | 55.1 | 157.5 |
| 24 | 2/12 | 343.8 | 280.1 | 413.8 | 3/15 | 130.9 | 119.8 | 142.8 | 4/7 | 114.4 | 62.1 | 168.8 |
| 25 | 2/13 | 420.2 | 340.0 | 505.9 | 3/16 | 142.6 | 131.5 | 154.4 | 4/8 | 125.2 | 69.6 | 178.5 |
| 26 | 2/14 | 511.3 | 422.4 | 625.3 | - | - | - | - | - | - | - | - |
| 27 | 2/15 | 619.0 | 510.4 | 754.9 | - | - | - | - | - | - | - | - |
| 28 | **2/16** | **744.8** | **613.6** | **905.1** | - | - | - | - | - | - | - | - |
| 29 | 2/17 | 889.7 | 733.5 | 1077.1 | - | - | - | - | - | - | - | - |
| 30 | 2/18 | 1053.9 | 875.1 | 1271.5 | - | - | - | - | - | - | - | - |
| 31 | 2/19 | 1236.7 | 1028.9 | 1480.6 | - | - | - | - | - | - | - | - |
| 32 | 2/20 | 1435.9 | 1211.1 | 1712.6 | - | - | - | - | - | - | - | - |
| 33 | 2/21 | 1647.8 | 1392.2 | 1937.2 | - | - | - | - | - | - | - | - |
| 34 | 2/22 | 1867.5 | 1596.3 | 2174.8 | - | - | - | - | - | - | - | - |
| 35 | 2/23 | 2089.1 | 1808.2 | 2408.1 | - | - | - | - | - | - | - | - |
| 36 | 2/24 | 2306.1 | 2003.9 | 2607.9 | - | - | - | - | - | - | - | - |
| 37 | 2/25 | 2512.4 | 2216.6 | 2811.0 | - | - | - | - | - | - | - | - |
| 38 | 2/26 | 2702.9 | 2417.3 | 2984.6 | - | - | - | - | - | - | - | - |
| 39 | 2/27 | 2873.7 | 2628.6 | 3157.2 | - | - | - | - | - | - | - | - |
| 40 | 2/28 | 3022.9 | 2792.6 | 3276.8 | - | - | - | - | - | - | - | - |

E: exposed state; I: infectious state; R: recovery state. CI: credible interval. Cumulated cases included one index case. Cases were confirmed in Japan and outside Japan on Diamond Princess cruise ship.

**Table C6. Results of COVID-19 cases under no vaccines protection were simulated by the Bayesian Markov Chain Monte Carlo (MCMC) method.**

|  | **No vaccines protection without NPIs** | | | | | | **No vaccines protection with NPIs since day 7** | | | | | |
| --- | --- | --- | --- | --- | --- | --- | --- | --- | --- | --- | --- | --- |
| **Day** | **detectable case**  **95% CI** | | | **final cases**  **95% CI** | | | **detectable case**  **(95% CI)** | | | **final cases**  **95% CI** | | |
| 1 | 1.1 | 1.1 | 1.1 | 1.8 | 1.6 | 1.9 | 1.1 | 1.1 | 1.1 | 1.8 | 1.6 | 1.9 |
| 2 | 1.3 | 1.2 | 1.3 | 2.5 | 2.2 | 2.9 | 1.3 | 1.2 | 1.3 | 2.5 | 2.2 | 2.8 |
| 3 | 1.5 | 1.4 | 1.7 | 3.4 | 2.9 | 3.9 | 1.5 | 1.4 | 1.7 | 3.4 | 2.9 | 3.9 |
| 4 | 1.9 | 1.7 | 2.2 | 4.4 | 3.6 | 5.2 | 1.9 | 1.7 | 2.2 | 4.4 | 3.5 | 5.2 |
| 5 | 2.5 | 2.1 | 2.8 | 5.6 | 4.5 | 6.9 | 2.5 | 2.1 | 2.8 | 5.6 | 4.4 | 6.8 |
| 6 | 3.1 | 2.6 | 3.7 | 7.1 | 5.4 | 8.9 | 3.1 | 2.6 | 3.7 | 7.1 | 5.3 | 8.8 |
| 7 | 4.0 | 3.2 | 4.9 | 9.0 | 6.6 | 11.7 | 4.0 | 3.1 | 4.8 | 9.0 | 6.5 | 11.5 |
| 8 | 5.0 | 3.9 | 6.4 | 11.3 | 8.1 | 15.1 | 4.9 | 3.8 | 6.1 | 9.8 | 7.0 | 12.5 |
| 9 | 6.4 | 4.8 | 8.3 | 14.3 | 9.8 | 19.6 | 5.8 | 4.4 | 7.3 | 10.7 | 7.5 | 13.7 |
| 10 | 8.0 | 5.9 | 10.8 | 17.9 | 11.9 | 25.4 | 6.7 | 5.0 | 8.6 | 11.7 | 8.2 | 15.3 |
| 11 | 10.1 | 7.1 | 14.1 | 22.6 | 14.4 | 32.9 | 7.7 | 5.6 | 9.9 | 12.9 | 8.9 | 17.2 |
| 12 | 12.8 | 8.7 | 18.3 | 28.4 | 17.4 | 42.6 | 8.7 | 6.3 | 11.4 | 14.2 | 9.6 | 19.3 |
| 13 | 16.1 | 9.9 | 23.1 | 35.7 | 21.0 | 55.1 | 9.8 | 6.9 | 12.9 | 15.6 | 10.2 | 21.6 |
| 14 | 20.2 | 11.9 | 29.8 | 44.8 | 25.1 | 70.9 | 10.9 | 7.6 | 14.6 | 17.1 | 11.1 | 24.6 |

CI: credible interval; NPIs: Non-pharmaceutical interventions

**Table C7. Results of COVID-19 cases under 10% vaccines protection were simulated by the Bayesian Markov Chain Monte Carlo (MCMC) method.**

|  | **10% vaccines protection without NPIs** | | | | | | **10% vaccines protection with NPIs since day 7** | | | | | |
| --- | --- | --- | --- | --- | --- | --- | --- | --- | --- | --- | --- | --- |
| **Day** | **detectable case**  **(95% CI)** | | | **final cases**  **95% CI** | | | **detectable case**  **(95% CI)** | | | **final cases**  **95% CI** | | |
| 1 | 1.1 | 1.0 | 1.1 | 1.7 | 1.6 | 1.8 | 1.1 | 1.0 | 1.1 | 1.7 | 1.6 | 1.8 |
| 2 | 1.2 | 1.2 | 1.3 | 2.4 | 2.1 | 2.7 | 1.2 | 1.2 | 1.3 | 2.4 | 2.1 | 2.6 |
| 3 | 1.5 | 1.4 | 1.6 | 3.1 | 2.7 | 3.6 | 1.5 | 1.4 | 1.6 | 3.1 | 2.6 | 3.5 |
| 4 | 1.8 | 1.7 | 2.0 | 3.9 | 3.3 | 4.7 | 1.8 | 1.7 | 2.0 | 3.9 | 3.2 | 4.6 |
| 5 | 2.3 | 2.0 | 2.6 | 5.0 | 4.0 | 6.1 | 2.3 | 2.0 | 2.6 | 5.0 | 4.0 | 6.0 |
| 6 | 2.9 | 2.4 | 3.4 | 6.2 | 4.8 | 7.7 | 2.8 | 2.4 | 3.3 | 6.2 | 4.7 | 7.6 |
| 7 | 3.5 | 2.9 | 4.3 | 7.7 | 5.8 | 9.8 | 3.5 | 2.9 | 4.2 | 7.7 | 5.7 | 9.7 |
| 8 | 4.4 | 3.5 | 5.5 | 9.5 | 6.9 | 12.5 | 4.3 | 3.4 | 5.2 | 8.3 | 6.0 | 10.4 |
| 9 | 5.5 | 4.2 | 7.0 | 11.7 | 8.3 | 15.9 | 5.0 | 3.9 | 6.3 | 9.0 | 6.6 | 11.5 |
| 10 | 6.8 | 5.1 | 9.0 | 14.5 | 9.9 | 20.1 | 5.8 | 4.4 | 7.3 | 9.8 | 7.0 | 12.6 |
| 11 | 8.4 | 6.1 | 11.4 | 17.9 | 11.7 | 25.5 | 6.6 | 5.0 | 8.4 | 10.7 | 7.6 | 14.0 |
| 12 | 10.4 | 7.2 | 14.5 | 22.0 | 13.9 | 32.4 | 7.3 | 5.4 | 9.4 | 11.6 | 8.0 | 15.4 |
| 13 | 12.8 | 8.2 | 17.9 | 27.1 | 16.5 | 40.9 | 8.2 | 5.9 | 10.6 | 12.6 | 8.5 | 17.1 |
| 14 | 15.8 | 9.7 | 22.7 | 33.4 | 19.5 | 51.7 | 9.0 | 6.5 | 11.9 | 13.7 | 9.2 | 19.3 |

CI: credible interval; NPIs: Non-pharmaceutical interventions**Table C8. Results of COVID-19 cases under 30% vaccines protection were simulated by the Bayesian Markov Chain Monte Carlo (MCMC) method.**

|  | **30% vaccines protection without NPIs** | | | | | | **30% vaccines protection with NPIs since day 7** | | | | | |
| --- | --- | --- | --- | --- | --- | --- | --- | --- | --- | --- | --- | --- |
| **Day** | **detectable case**  **(95% CI)** | | | **final cases**  **95% CI** | | | **detectable case**  **(95% CI)** | | | **final cases**  **95% CI** | | |
| 1 | 1.0 | 1.0 | 1.1 | 1.5 | 1.4 | 1.6 | 1.0 | 1.0 | 1.1 | 1.5 | 1.4 | 1.6 |
| 2 | 1.2 | 1.1 | 1.2 | 2.0 | 1.9 | 2.3 | 1.2 | 1.1 | 1.2 | 2.0 | 1.8 | 2.2 |
| 3 | 1.4 | 1.3 | 1.5 | 2.6 | 2.3 | 2.9 | 1.4 | 1.3 | 1.5 | 2.6 | 2.2 | 2.9 |
| 4 | 1.6 | 1.5 | 1.8 | 3.2 | 2.7 | 3.7 | 1.6 | 1.5 | 1.8 | 3.2 | 2.7 | 3.6 |
| 5 | 1.9 | 1.8 | 2.2 | 3.8 | 3.2 | 4.5 | 1.9 | 1.7 | 2.2 | 3.8 | 3.2 | 4.5 |
| 6 | 2.3 | 2.0 | 2.7 | 4.6 | 3.7 | 5.5 | 2.3 | 2.0 | 2.7 | 4.6 | 3.6 | 5.5 |
| 7 | 2.8 | 2.4 | 3.3 | 5.4 | 4.3 | 6.7 | 2.8 | 2.3 | 3.2 | 5.4 | 4.2 | 6.7 |
| 8 | 3.3 | 2.8 | 4.0 | 6.5 | 5.0 | 8.2 | 3.3 | 2.7 | 3.9 | 5.8 | 4.4 | 7.1 |
| 9 | 4.0 | 3.2 | 4.9 | 7.7 | 5.7 | 10.0 | 3.7 | 3.0 | 4.5 | 6.2 | 4.8 | 7.7 |
| 10 | 4.7 | 3.7 | 6.0 | 9.1 | 6.6 | 12.1 | 4.2 | 3.3 | 5.1 | 6.6 | 5.0 | 8.2 |
| 11 | 5.6 | 4.3 | 7.3 | 10.8 | 7.6 | 14.7 | 4.7 | 3.7 | 5.8 | 7.1 | 5.4 | 9.0 |
| 12 | 6.7 | 5.0 | 8.9 | 12.8 | 8.7 | 17.9 | 5.1 | 4.0 | 6.3 | 7.5 | 5.6 | 9.6 |
| 13 | 7.9 | 5.5 | 10.6 | 15.1 | 10.0 | 21.7 | 5.6 | 4.3 | 6.9 | 8.0 | 5.8 | 10.4 |
| 14 | 9.4 | 6.3 | 12.8 | 17.8 | 11.2 | 26.0 | 6.0 | 4.6 | 7.6 | 8.6 | 6.0 | 11.3 |

CI: credible interval; NPIs: Non-pharmaceutical interventions

**Table C9. Results of COVID-19 cases under 50% vaccines protection were simulated by the Bayesian Markov Chain Monte Carlo (MCMC) method.**

|  | **50% vaccines protection without NPIs** | | | | | | **50% vaccines protection with NPIs since day 7** | | | | | |
| --- | --- | --- | --- | --- | --- | --- | --- | --- | --- | --- | --- | --- |
| **Day** | **detectable case**  **(95% CI)** | | | **final cases**  **95% CI** | | | **detectable case**  **(95% CI)** | | | **final cases**  **95% CI** | | |
| 1 | 1.0 | 1.0 | 1.0 | 1.4 | 1.3 | 1.5 | 1.0 | 1.0 | 1.0 | 1.4 | 1.3 | 1.5 |
| 2 | 1.1 | 1.1 | 1.2 | 1.7 | 1.6 | 1.9 | 1.1 | 1.1 | 1.2 | 1.7 | 1.6 | 1.9 |
| 3 | 1.3 | 1.2 | 1.3 | 2.1 | 1.9 | 2.3 | 1.3 | 1.2 | 1.3 | 2.1 | 1.9 | 2.3 |
| 4 | 1.4 | 1.3 | 1.5 | 2.4 | 2.2 | 2.8 | 1.4 | 1.3 | 1.5 | 2.4 | 2.1 | 2.7 |
| 5 | 1.6 | 1.5 | 1.8 | 2.8 | 2.5 | 3.3 | 1.6 | 1.5 | 1.8 | 2.8 | 2.4 | 3.2 |
| 6 | 1.9 | 1.7 | 2.1 | 3.3 | 2.7 | 3.8 | 1.9 | 1.7 | 2.1 | 3.3 | 2.7 | 3.8 |
| 7 | 2.2 | 1.9 | 2.4 | 3.7 | 3.1 | 4.4 | 2.2 | 1.9 | 2.4 | 3.7 | 3.1 | 4.4 |
| 8 | 2.5 | 2.1 | 2.9 | 4.2 | 3.4 | 5.2 | 2.4 | 2.1 | 2.8 | 3.9 | 3.2 | 4.6 |
| 9 | 2.8 | 2.4 | 3.3 | 4.8 | 3.8 | 5.9 | 2.7 | 2.3 | 3.1 | 4.1 | 3.3 | 4.8 |
| 10 | 3.2 | 2.7 | 3.9 | 5.4 | 4.2 | 6.9 | 3.0 | 2.5 | 3.5 | 4.3 | 3.5 | 5.2 |
| 11 | 3.7 | 3.0 | 4.5 | 6.1 | 4.6 | 7.9 | 3.2 | 2.7 | 3.8 | 4.5 | 3.6 | 5.4 |
| 12 | 4.2 | 3.3 | 5.2 | 6.9 | 5.1 | 9.1 | 3.4 | 2.9 | 4.1 | 4.7 | 3.8 | 5.7 |
| 13 | 4.7 | 3.7 | 6.1 | 7.8 | 5.6 | 10.5 | 3.7 | 3.0 | 4.4 | 4.9 | 3.8 | 6.0 |
| 14 | 5.3 | 4.0 | 6.9 | 8.8 | 6.2 | 12.0 | 3.9 | 3.2 | 4.7 | 5.1 | 3.9 | 6.4 |

CI: credible interval; NPIs: Non-pharmaceutical interventions

**Table C10. Results of COVID-19 cases under 70% vaccines protection were simulated by the Bayesian Markov Chain Monte Carlo (MCMC) method.**

|  | **70% vaccines protection without NPIs** | | | | | | **70% vaccines protection with NPIs since day 7** | | | | | |
| --- | --- | --- | --- | --- | --- | --- | --- | --- | --- | --- | --- | --- |
| **Day** | **detectable case**  **(95% CI)** | | | **final cases**  **95% CI** | | | **detectable case**  **(95% CI)** | | | **final cases**  **95% CI** | | |
| 1 | 1.0 | 1.0 | 1.0 | 1.2 | 1.2 | 1.3 | 1.0 | 1.0 | 1.0 | 1.2 | 1.2 | 1.3 |
| 2 | 1.1 | 1.1 | 1.1 | 1.4 | 1.4 | 1.5 | 1.1 | 1.1 | 1.1 | 1.4 | 1.3 | 1.5 |
| 3 | 1.2 | 1.1 | 1.2 | 1.6 | 1.5 | 1.8 | 1.2 | 1.1 | 1.2 | 1.6 | 1.5 | 1.7 |
| 4 | 1.3 | 1.2 | 1.3 | 1.8 | 1.7 | 2.0 | 1.3 | 1.2 | 1.3 | 1.8 | 1.6 | 2.0 |
| 5 | 1.4 | 1.3 | 1.4 | 2.0 | 1.8 | 2.2 | 1.4 | 1.3 | 1.4 | 2.0 | 1.8 | 2.2 |
| 6 | 1.5 | 1.4 | 1.6 | 2.2 | 2.0 | 2.5 | 1.5 | 1.4 | 1.6 | 2.2 | 1.9 | 2.4 |
| 7 | 1.6 | 1.5 | 1.8 | 2.4 | 2.1 | 2.7 | 1.6 | 1.5 | 1.8 | 2.4 | 2.1 | 2.7 |
| 8 | 1.8 | 1.6 | 2.0 | 2.6 | 2.2 | 3.0 | 1.8 | 1.6 | 1.9 | 2.5 | 2.1 | 2.8 |
| 9 | 1.9 | 1.7 | 2.2 | 2.8 | 2.4 | 3.3 | 1.9 | 1.7 | 2.1 | 2.5 | 2.2 | 2.9 |
| 10 | 2.1 | 1.9 | 2.4 | 3.0 | 2.5 | 3.6 | 2.0 | 1.8 | 2.2 | 2.6 | 2.2 | 2.9 |
| 11 | 2.3 | 2.0 | 2.6 | 3.2 | 2.7 | 3.9 | 2.1 | 1.9 | 2.4 | 2.7 | 2.3 | 3.0 |
| 12 | 2.5 | 2.1 | 2.9 | 3.5 | 2.8 | 4.2 | 2.2 | 1.9 | 2.5 | 2.7 | 2.4 | 3.1 |
| 13 | 2.7 | 2.3 | 3.1 | 3.7 | 3.0 | 4.6 | 2.3 | 2.0 | 2.6 | 2.8 | 2.4 | 3.2 |
| 14 | 2.9 | 2.4 | 3.4 | 4.0 | 3.2 | 4.9 | 2.4 | 2.1 | 2.7 | 2.9 | 2.4 | 3.3 |

CI: credible interval; NPIs: Non-pharmaceutical interventions

**Table C11. Results of COVID-19 cases under 90% vaccines protection were simulated by the Bayesian Markov Chain Monte Carlo (MCMC) method.**

|  | **90% vaccines protection without NPIs** | | | | | | **90% vaccines protection with NPIs since day 7** | | | | | |
| --- | --- | --- | --- | --- | --- | --- | --- | --- | --- | --- | --- | --- |
| **Day** | **detectable case**  **(95% CI)** | | | **final cases**  **95% CI** | | | **detectable case**  **(95% CI)** | | | **final cases**  **95% CI** | | |
| 1 | 1.0 | 1.0 | 1.0 | 1.1 | 1.1 | 1.1 | 1.0 | 1.0 | 1.0 | 1.1 | 1.1 | 1.1 |
| 2 | 1.0 | 1.0 | 1.0 | 1.1 | 1.1 | 1.2 | 1.0 | 1.0 | 1.0 | 1.1 | 1.1 | 1.2 |
| 3 | 1.0 | 1.0 | 1.1 | 1.2 | 1.2 | 1.2 | 1.0 | 1.0 | 1.1 | 1.2 | 1.2 | 1.2 |
| 4 | 1.1 | 1.1 | 1.1 | 1.3 | 1.2 | 1.3 | 1.1 | 1.1 | 1.1 | 1.3 | 1.2 | 1.3 |
| 5 | 1.1 | 1.1 | 1.1 | 1.3 | 1.2 | 1.4 | 1.1 | 1.1 | 1.1 | 1.3 | 1.2 | 1.4 |
| 6 | 1.2 | 1.1 | 1.2 | 1.3 | 1.3 | 1.4 | 1.2 | 1.1 | 1.2 | 1.3 | 1.3 | 1.4 |
| 7 | 1.2 | 1.2 | 1.2 | 1.4 | 1.3 | 1.5 | 1.2 | 1.2 | 1.2 | 1.4 | 1.3 | 1.5 |
| 8 | 1.2 | 1.2 | 1.3 | 1.4 | 1.3 | 1.5 | 1.2 | 1.2 | 1.3 | 1.4 | 1.3 | 1.5 |
| 9 | 1.3 | 1.2 | 1.3 | 1.5 | 1.4 | 1.6 | 1.3 | 1.2 | 1.3 | 1.4 | 1.3 | 1.5 |
| 10 | 1.3 | 1.2 | 1.4 | 1.5 | 1.4 | 1.6 | 1.3 | 1.2 | 1.3 | 1.4 | 1.3 | 1.5 |
| 11 | 1.3 | 1.3 | 1.4 | 1.5 | 1.4 | 1.6 | 1.3 | 1.3 | 1.4 | 1.4 | 1.4 | 1.5 |
| 12 | 1.4 | 1.3 | 1.5 | 1.6 | 1.4 | 1.7 | 1.3 | 1.3 | 1.4 | 1.4 | 1.4 | 1.5 |
| 13 | 1.4 | 1.3 | 1.5 | 1.6 | 1.5 | 1.7 | 1.4 | 1.3 | 1.4 | 1.5 | 1.4 | 1.5 |
| 14 | 1.4 | 1.4 | 1.5 | 1.6 | 1.5 | 1.8 | 1.4 | 1.3 | 1.4 | 1.5 | 1.4 | 1.6 |

CI: credible interval; NPIs: Non-pharmaceutical interventions

**Table C12. Results of COVID-19 cases under different vaccine protection with or without NPIs when cases were found on the 7th day of voyage during 14 days voyage were simulated by the Bayesian Markov Chain Monte Carlo (MCMC) method.**

|  | **One infectious case with COVID-19 boards the cruise ship** | | | | | | **Five infectious cases with COVID-19 boards the cruise ship** | | | | | |
| --- | --- | --- | --- | --- | --- | --- | --- | --- | --- | --- | --- | --- |
|  | **No NPIs** | | | **NPIs since the 7th day of voyage** | | | **No NPIs** | | | **NPIs since the 7th day of voyage** | | |
| **Vaccine protection** | **Total Cases** | **(95% CI)** | | **Total Cases** | **(95% CI)** | | **Total Cases** | **(95% CI)** | | **Total Cases** | **(95% CI)** | |
| 0% | 44.8 | 25.1 | 70.9 | 17.1 | 11.1 | 24.6 | 215.2 | 123.1 | 334.3 | 84.0 | 54.9 | 120.6 |
| 10% | 33.4 | 19.5 | 51.7 | 13.7 | 9.2 | 19.3 | 162.3 | 48.0 | 110.6 | 67.7 | 45.8 | 95.1 |
| 20% | 24.6 | 15.0 | 37.2 | 10.9 | 7.6 | 14.9 | 120.4 | 74.2 | 180.2 | 54.0 | 37.7 | 73.7 |
| 30% | 17.8 | 11.2 | 26.0 | 8.6 | 6.0 | 11.3 | 87.7 | 57.0 | 128.6 | 42.6 | 30.0 | 56.0 |
| 40% | 9.5 | 8.5 | 18.0 | 6.7 | 4.9 | 8.6 | 62.7 | 42.1 | 88.6 | 33.2 | 24.5 | 42.5 |
| 50% | 8.8 | 6.2 | 12.0 | 5.1 | 3.9 | 6.4 | 43.8 | 30.7 | 59.4 | 25.5 | 19.7 | 31.7 |
| 60% | 6.0 | 4.4 | 7.8 | 3.9 | 3.1 | 4.7 | 29.9 | 22.2 | 38.9 | 19.3 | 15.5 | 23.2 |
| 70% | 4 | 3.2 | 4.9 | 2.9 | 2.4 | 3.3 | 19.9 | 15.7 | 24.6 | 14.3 | 12.1 | 16.6 |
| 80% | 2.8 | 2.2 | 3 | 2.1 | 1.9 | 2.3 | 12.9 | 10.9 | 15.0 | 10.4 | 9.3 | 11.5 |
| 90% | 1.6 | 1.5 | 1.8 | 1.5 | 1.4 | 1.6 | 8.1 | 7.5 | 8.8 | 7.3 | 6.9 | 7.8 |

CI: credible interval; NPIs: Non-pharmaceutical interventions

**References:**

(1) **Oran DP, Topol EJ.** Prevalence of Asymptomatic SARS-CoV-2 Infection: A Narrative Review. *Ann Intern Med* 2020.

(2) Peniston B. The Battle of USS Theodore Roosevelt: a Timeline. In, 2020.

(3) **Kasper MR, et al.** An Outbreak of Covid-19 on an Aircraft Carrier. *The New England Journal of Medicine* 2020; **383**(25): 2417-2426.

(4) **de Laval F, et al.** Investigation of a COVID-19 outbreak on the Charles de Gaulle aircraft carrier, March to April 2020: a retrospective cohort study. *Euro Surveillance* 2022; **27**(21).

(5) **Chen CM, et al.** Containing COVID-19 Among 627,386 Persons in Contact With the Diamond Princess Cruise Ship Passengers Who Disembarked in Taiwan: Big Data Analytics. *Journal of Medical Internet Research* 2020; **22**(5): e19540.

(6) **Kakimoto K, et al.** Initial Investigation of Transmission of COVID-19 Among Crew Members During Quarantine of a Cruise Ship - Yokohama, Japan, February 2020. *MMWR Morbidity and Mortality Weekly Report* 2020; **69**(11): 312-313.

(7) **Lai CC, et al.** The Bayesian Susceptible-Exposed-Infected-Recovered model for the outbreak of COVID-19 on the Diamond Princess Cruise Ship. *Stochastic Environmental Research and Risk Assessment* 2021: 1-15.

(8) **Moriarty LF, et al.** Public Health Responses to COVID-19 Outbreaks on Cruise Ships - Worldwide, February-March 2020. *MMWR Morbidity and Mortality Weekly Report* 2020; **69**(12): 347-352.

(9) Anon. COVID-19 pandemic on Grand Princess. In, 2020.

(10) Tate C. Some Grand Princess cruise passengers waited weeks for coronavirus test results, or never got tested. In: USA today, 2020.

(11) **Ing AJ, Cocks C, Green JP.** COVID-19: in the footsteps of Ernest Shackleton. *Thorax* 2020.

(12) **Li Q, et al.** Early Transmission Dynamics in Wuhan, China, of Novel Coronavirus-Infected Pneumonia. *The New England Journal of Medicine* 2020; **382**(13): 1199-1207.

(13) **Breiman L.** Random Forests. *Machine Learning* 2001; **45**: 5-32.

(14) **Guan WJ, et al.** Clinical Characteristics of Coronavirus Disease 2019 in China. *The New England Journal of Medicine* 2020; **382**(18): 1708-1720.

(15) **Wu Y, et al.** Incubation Period of COVID-19 Caused by Unique SARS-CoV-2 Strains: A Systematic Review and Meta-analysis. The Journal of the American Medical Association, JAMA Network Open 2022; **5**(8): e2228008.
